# mTORC1 regulates PTHrP to coordinate chondrocyte growth, proliferation and differentiation

**DOI:** 10.1038/ncomms11151

**Published:** 2016-04-04

**Authors:** Bo Yan, Zhongmin Zhang, Dadi Jin, Chen Cai, Chunhong Jia, Wen Liu, Ting Wang, Shengfa Li, Haiyan Zhang, Bin Huang, Pinglin Lai, Hua Wang, Anling Liu, Chun Zeng, Daozhang Cai, Yu Jiang, Xiaochun Bai

**Affiliations:** 1 Academy of Orthopedics, Guangdong Province, The Third Affiliated Hospital, Southern Medical University, Guangzhou 510630, China; 2 Department of Orthopedics, Zhujiang Hospital, Southern Medical University, Guangzhou 510282, China; 3 State Key Laboratory of Organ Failure Research, Department of Cell Biology, School of Basic Medical Sciences, Southern Medical University, Guangzhou 510515, China; 4 Department of Pharmacology and Chemical Biology, University of Pittsburgh School of Medicine, Pittsburgh, Pennsylvania 15213, USA

## Abstract

Precise coordination of cell growth, proliferation and differentiation is essential for the development of multicellular organisms. Here, we report that although the mechanistic target of rapamycin complex 1 (mTORC1) activity is required for chondrocyte growth and proliferation, its inactivation is essential for chondrocyte differentiation. Hyperactivation of mTORC1 via *TSC1* gene deletion in chondrocytes causes uncoupling of the normal proliferation and differentiation programme within the growth plate, resulting in uncontrolled cell proliferation, and blockage of differentiation and chondrodysplasia in mice. Rapamycin promotes chondrocyte differentiation and restores these defects in mutant mice. Mechanistically, mTORC1 downstream kinase S6K1 interacts with and phosphorylates Gli2, and releases Gli2 from SuFu binding, resulting in nuclear translocation of Gli2 and transcription of parathyroid hormone-related peptide (PTHrP), a key regulator of bone development. Our findings demonstrate that dynamically controlled mTORC1 activity is crucial to coordinate chondrocyte proliferation and differentiation partially through regulating Gli2/PTHrP during endochondral bone development.

## Abstract (web summary)

mTORC1 is crucial for chondrocyte proliferation and bone growth, but the downstream signalling is not clear. Here, the authors use rapamycin and chondrocyte-specific Tsc1 knockout mice to show that S6K1 can cause nuclear accumulation of Gli2, thus driving PTHrP expression and preventing terminal differentiation of prehypertrophic chondrocytes.

## Graphical abstract

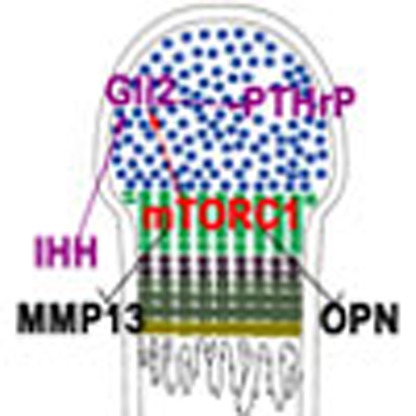

During development of multicellular organisms, cells proliferate for a defined length of time before functional differentiation is initiated. Increase in cell size, proliferation, differentiation and apoptosis are features of all cells that have not terminally differentiated^1^,^2^. Although substantial coupling between regulation of cell growth, proliferation and differentiation has been demonstrated, the mechanisms underlying precise coordination of cell proliferation and differentiation to ensure production of the proper numbers of differentiated cells at the appropriate times remain largely unknown^3^,^4^,^5^.

Mechanistic target of rapamycin (mTOR) is a conserved protein kinase that forms two distinct functional complexes, mTOR complex 1 (mTORC1) and complex 2 (mTORC2). mTORC1 uniquely contains raptor and is the target of rapamycin that controls protein synthesis through phosphorylation of translational regulators, eukaryotic initiation factor 4E–binding protein-1 (4E-BP1) and S6 kinase 1 (S6K1). In response to nutrients, growth factors, energy and stress, mTORC1 is activated by two families of ras-related small guanosine triphosphatases (GTPase), Rheb and Rags^6^,^7^,^8^. GTP-bound (active) Rheb is suppressed by tuberous sclerosis complex 1/2 (TSC1/2), a functional complex displaying GTPase-activating protein (GAP) activity towards Rheb. Loss of TSC1/2 triggers constitutive mTORC1 activation^9^. Although mTORC1 has been established as a central regulator of cell growth, proliferation and metabolism, its specific functions in cell differentiation and development are less well understood^5^,^10^,^11^. Moreover, the changes in mTORC1 activity during development to coordinate cell proliferation and differentiation and the underlying pathways remain to be established.

Endochondral bone formation depends on a highly coordinated programme of proliferation, differentiation and maturation through permanent withdrawal from the cell cycle, hypertrophy and terminal differentiation of chondrocytes within the mammalian growth plate. Disruption of this programme results in chondrodysplasia or malformed skeleton, thus presenting a well-defined and clinically important model for investigation of cell proliferation and differentiation^12^,^13^. The process is initiated with condensation of mesenchymal cells within the embryonic limb bud. Subsequently, cells at the core of mesenchymal condensation differentiate into chondrocytes, which secrete cartilage matrix rich in type II collagen and several proteoglycans, such as aggrecan. Following initial proliferation, chondrocytes become increasingly organized into morphologically distinct domains. At either end of the template, proliferating chondrocytes exhibit rounded morphology (round chondrocytes), but become flattened and stacked in columns (columnar chondrocytes) towards the middle of the cartilage rod. Chondrocytes in the middle of the primordial template exit the cell cycle, begin to differentiate and undergo hypertrophy. Hypertrophy, the main contributor to skeletal growth, is divided into three sequential phases, specifically, prehypertrophy, hypertrophy and terminal hypertrophy. Hypertrophic chondrocytes suppress production of type II collagen while beginning to secrete type X collagen. Terminal hypertrophic chondrocytes also release factors responsible for the breakdown of matrix and calcification, including vascular endothelial growth factor, matrix metalloproteinase 13 (MMP-13), bone morphogenetic protein-2, osteopontin (OPN) and osteocalcin, and eventually undergo apoptosis, followed by invasion of blood vessels from the perichondrium. The invading vasculature not only triggers resorption of hypertrophic cartilage matrix and formation of the bone marrow cavity, but also recruits osteoblast and osteoclast precursors that produce cancellous bone within the marrow cavity, and cartilage is eventually replaced by mineralized bone deposits^14^,^15^,^16^. This programme is regulated by a complex network of molecules, including Indian hedgehog (IHH), parathyroid hormone-related peptide (PTHrP), bone morphogenetic proteins, fibroblast growth factors and the respective receptors, and interactions between cells and the extracellular matrix^17^,^18^,^19^.

Although mTORC1 signalling has been shown to be essential for chondrocyte proliferation and bone development^20^,^21^,^22^, its specific role in chondrocyte differentiation and the underlying mechanisms are unclear. Here, we show that mTORC1 activity declines during the progression of chondrocyte differentiation *in vitro* and *in vivo*. Activation of mTORC1 in chondrocytes promotes growth and proliferation, but stimulates the transcription factor glioma-associated oncogene homologue 2 (Gli2) and PTHrP expression to prevent hypertrophic chondrocyte terminal differentiation. Our findings show that activation of Gli2 by mTORC1 is required for PTHrP transcription and that dynamic regulation of mTORC1 activity is crucial to coordinate chondrocyte growth, proliferation and differentiation during endochondral bone development.

## Results

### mTORC1 activity is altered during chondrocyte differentiation

**Figure 1:**
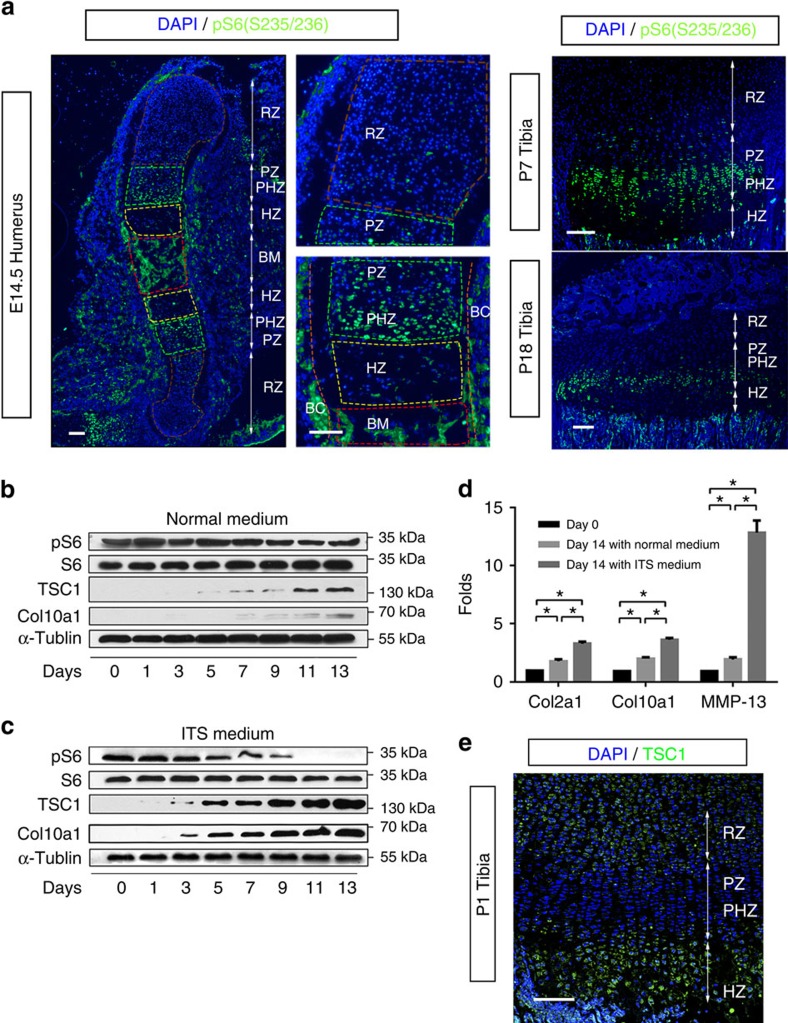
Dynamic changes of mTORC1 activity during chondrocyte differentiation (**a**) pS6 (S235/236) immunofluorescence in longitudinal frozen sections of mouse humerus at E14.5 or tibias at P7/P18. pS6, green; DNA, blue. Higher magnification is shown on the right. RZ, resting chondrocyte zone; PZ, proliferative chondrocyte zone; HZ, hypertrophic chondrocyte zone; PHZ, prehypertrophic chondrocyte zone; BM, bone marrow cavity; BC, bone collar. Scale bar, 100 μm. (**b**) Western blot of primary cultured chondrocytes isolated from C57BL/6 mice. Lysates were obtained from chondrocytes cultured in growth medium at different time points after confluence. (**c**) Western blot of primary chondrocytes culturedin ITS differentiation medium. Lysates were obtained at different time points after differentiation induction. (**d**) qPCR analysis of chondrocytes cultured in growth or ITS medium on days 0 and 14. ITS, insulin-transferrin-selenium medium; One-way ANOVA (analysis of variance) and Dunnett's multiple comparison test, *P<0.05, *n*=5; Error bars indicate s.d. (**e**) TSC1 immunofluorescence in longitudinal frozen sections of mouse tibias at P1. TSC1, green; DNA, blue. Scale bar, 100 μm.

To investigate the role of mTORC1 in chondrocyte proliferation and differentiation, we initially examined its activity in the growth plate of developing long bones. Phosphorylation of S6 (Ser235/236), an established readout for mTORC1 signalling, was detected at low levels among mesenchymal cells and round chondrocytes of humerus at days E14.5 (Fig. 1a). S6 phosphorylation was evident in proliferating columnar and prehypertrophic chondrocytes, but markedly reduced upon commitment to the hypertrophic chondrocyte differentiation programme, resulting in little staining within much of the hypertrophic region (Fig. 1a).

We next determined whether mTORC1 activity is dynamically altered during the process of proliferation and differentiation in cultured chondrocytes. High pS6 level (Ser 235/236) was maintained upon culture of chondrocytes in growth medium (Fig. 1b). However, when these cells were induced to differentiate, pS6 (Ser235/236) began to decline on day 3 after differentiation, decreased by 50% on day 7 and completely disappeared by day 11 (Fig. 1c). Chondrocyte differentiation was confirmed via assessment of collagen II, collagen X and MMP-13 expression (Fig. 1c,d). Interestingly, expression of TSC1, an upstream negative regulator of mTORC1 signalling, was enhanced upon chondrocyte differentiation *in vitro* (Fig. 1c) and negatively correlated with mTORC1 activity (pS6) in growth plate of developing bones (Fig. 1e). These results collectively demonstrate that TSC1 expression and mTORC1 activity changes dynamically during chondrocyte proliferation and differentiation, both *in vitro* and *in vivo.*

### Inhibition of mTORC1 promotes chondrocyte differentiation

**Figure 2:**
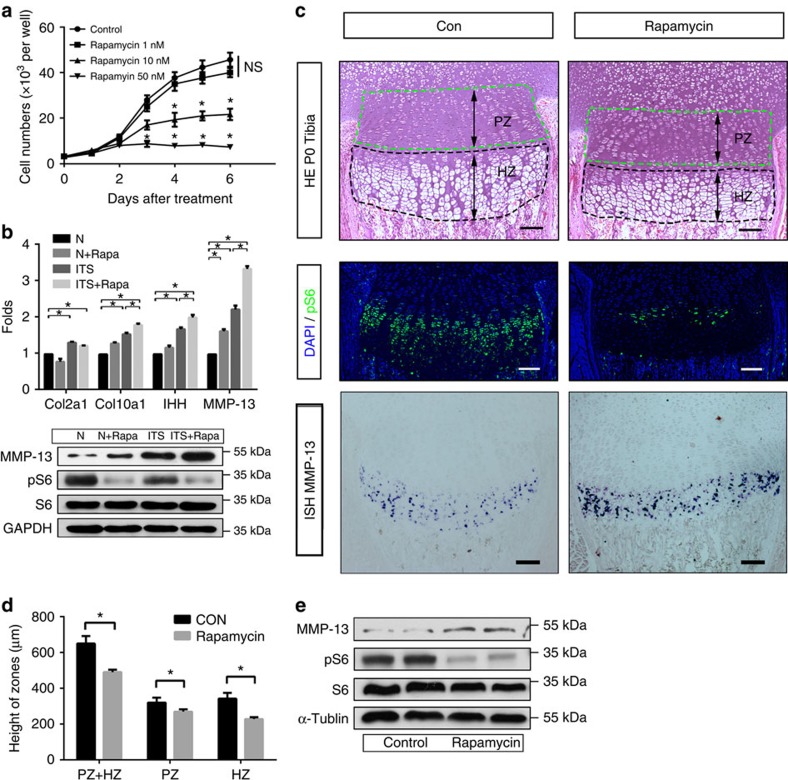
Inhibition of mTORC1 prevents chondrocyte proliferation while promoting differentiation. (**a**) CCK8 proliferation assay of chondrocytes cultured in normal growth medium or with 1, 10 or 50 nM rapamycin treatment. OD_450_values were converted to cell numbers. One-way analysis of variance (ANOVA) and Dunnett's multiple comparison test; NS, not significant, *P<0.05, *n*=5; Error bars indicate s.d. (**b**) qPCR and western blot analysis of primary chondrocytes cultured in growth medium or ITS medium. Cells were treated with or without rapamycin from day 6 and were harvested on day 14 after confluence. One-way ANOVA and Dunnett's multiple comparison test, *P<0.05, *n*≥3; Error bars indicate s.d. (**c**) HE staining, pS6 immunofluorescence and MMP-13 *in situ* mRNA analysis of Tibia sections of mice at P0 whose maternal mice receiving a daily intraperitoneal injection of rapamycin (1.5 mg kg^−1^ per day) or 0.9% saline for 3 days before sacrifice. Scale bar, 100 μm. PZ, proliferative chondrocyte zone; HZ, hypertrophic chondrocyte zone. (**d**) Quantification of Tibia PZ and HZ length in P0 mice receiving rapamycin. Student's *t*-test, *P<0.05, *n*≥3; Error bars indicate s.d. (**e**) Western blot of pure cartilage tissues harvested from long bone mice receiving rapamycin.

The observed dynamic changes in mTORC1 activity suggest different roles at various proliferation and differentiation stages. Upon treatment with the mTORC1-specific inhibitor, rapamycin, chondrocyte proliferation was suppressed in a dose-dependent manner (Fig. 2a), indicating an essential role of mTORC1 in this process. Next, we determined whether disruption of mTORC1 activity affects chondrocyte differentiation. Cultured chondrocytes at early and middle differentiation stages (days 1 and 6) were treated with rapamycin to inhibit mTORC1. Interestingly, rapamycin added on day 6 promoted terminal differentiation of chondrocytes, as evident from enhanced collagen X and MMP-13 expression (Fig. 2b). However, upon addition of rapamcyin on day 1, differentiation was inhibited (Supplementary Fig. 1a,b). The effects of rapamycin on chondrocyte proliferation and differentiation were further examined *in vivo*. Mice treated with rapamycin exhibited thinner growth plates and reduced chondrocytes at all stages, with lower proliferating columnar and prehypertrophic chondrocytes (Fig. 2c–e). These results suggest that activated mTORC1 is required for chondrocyte proliferation and early differentiation, while terminal differentiation requires low mTORC1 activity. Dynamic regulation of mTORC1 activity appears essential for the coordination of chondrocyte proliferation and differentiation.

### Loss of TSC1 in chondrocytes causes chondrodysplasia

**Figure 3:**
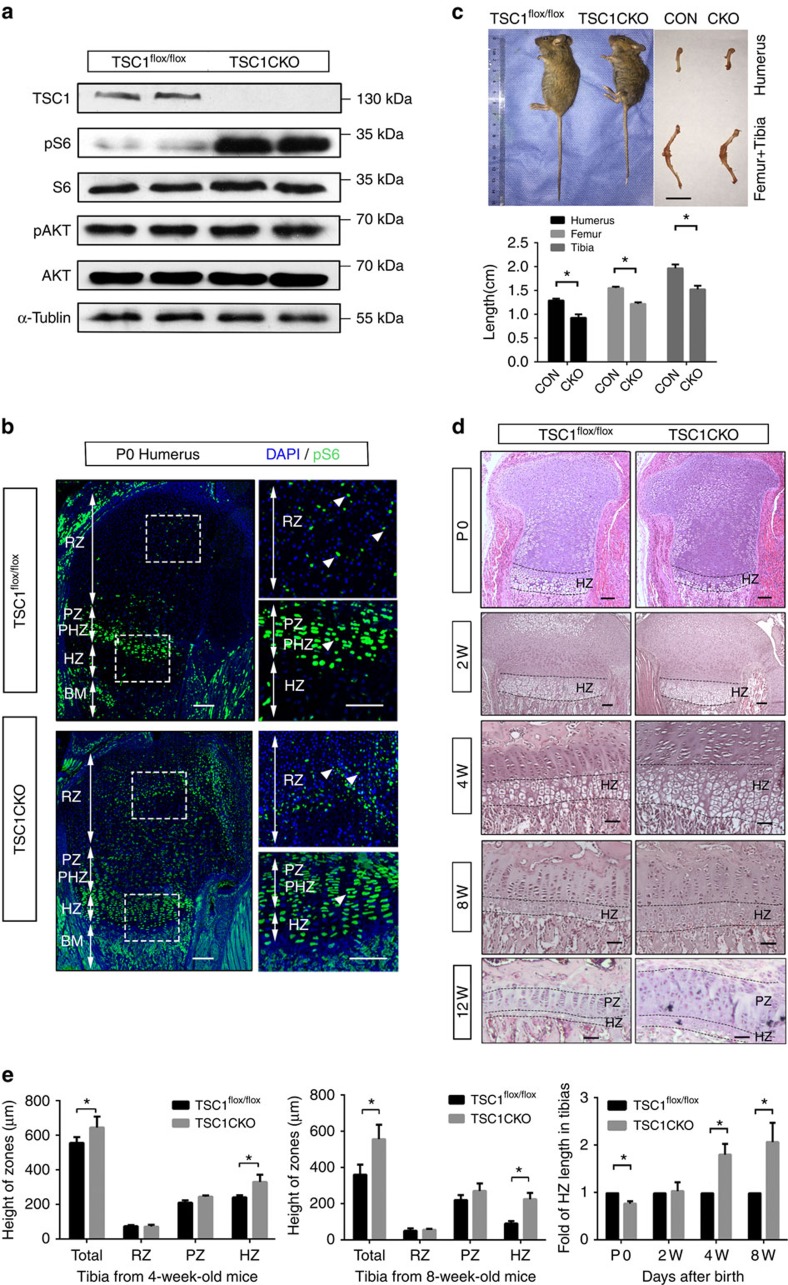
Activation of mTORC1 in chondrocytes causes chondrodysplasia and dwarfness in mice. (**a**) Western blot of pure cartilage tissues harvested from rib or long bone of P0 TSC1^flox/flox^ and TSC1CKO mice. (**b**) pS6 immunofluorescence analysis of longitudinal frozen sections of control and TSC1CKO mouse humerus at P0. Boxed areas are represented by higher magnification below. White triangles show the pS6 positive chondrocytes. Scale bar, 200 μm. (**c**) Image of 8-week-old TSC1CKO mice and humerus, femur and tibia. Scale bar, 1 cm. Student's *t*-test, *P<0.05, *n*=5; Error bars indicate s.d. (**d**) HE staining of tibia from 0-, 2-, 4-, 8- and 12-week-old mice. Upper lines specify where hypertrophic differentiation begins and lower lines show cartilage bone junctions. Scale bar, 100 μm. (**e**) Quantification of RZ, PZ and HZ lengths in 4- and 8-week-old TSC1CKO mice. Quantification of HZ length in tibia from 2-, 4- and 8-week-old mice. Student's *t*-test, *P<0.05, *n*≥3; Error bars indicate s.d.

In contrast to our findings, two previous reports found no effect on 5-Bromo-2-deoxyUridine (BrdU) incorporation in rapamycin-treated metatarsal cultures^23^ and in mouse mutants deficient for raptor or mTOR (Prx1-cre) in chondrocytes^20^. To gain insights into the specific role of activated mTORC1 in chondrocyte proliferation and endochondral bone development, we generated mice with *TSC1* gene deletions in chondrocytes (TSC1CKO) by crossing TSC1^flox/flox^ mice with Col2a1-cre mice (Supplementary Fig. 2a). To confirm recombination in chondrocytes, pure cartilage tissues were obtained from newborn TSC1CKO mice and littermates under the microscope. The deleted allele was amplified by PCR from tissue samples of TSC1CKO mice. (Supplementary Fig. 2b). As expected, TSC1 was specifically observed in wild-type (WT) littermates, but not TSC1CKO mice (Fig. 3a). Accordingly, specific enhancement of pS6(Ser235/236), but not pAkt (Ser473), in cartilage was observed in CKO mice (Fig. 3a,b).

Although TSC1CKO mice were born with normal Mendelian ratios and showed no differences in body length and weight at birth compared with their littermates, they displayed a progressive delay in body length and weight gain, and stopped increasing in terms of body length and weight at 5 weeks after birth (Supplementary Fig. 3a,b). The femur, humerus, tibia and spinal column of TSC1CKO mice were 20–25% shorter than those of control mice (Fig. 3c). The majority of TSC1CKO mice died before 15 weeks of age, possibly as a result of pneumonia. X-rays revealed significant pathological thoracic lordosis with severe rib wavy-like dysplasia in 3-month-old TSC1CKO mice, resulting in a considerably smaller thoracic volume relative to that in control littermates (Supplementary Fig. 3c,d). Histological analysis of humerus and tibia from P0, 2, 4 and 8-week-old mice showed reduced hypertrophic chondrocytes at P0, and subsequent postnatal progressive expansion of chondrocytes at all stages in TSC1CKO mice, which was most evident in hypertrophic chondrocytes, resulting in a significantly thicker growth plate compared to control mice (Fig. 3d,e). Based on these results, we propose that loss of *TSC1* delays postnatal endochondral bone development and causes chondrodysplasia and dwarfness in mice.

### Activation of mTORC1 prevents chondrocyte maturation

**Figure 4:**
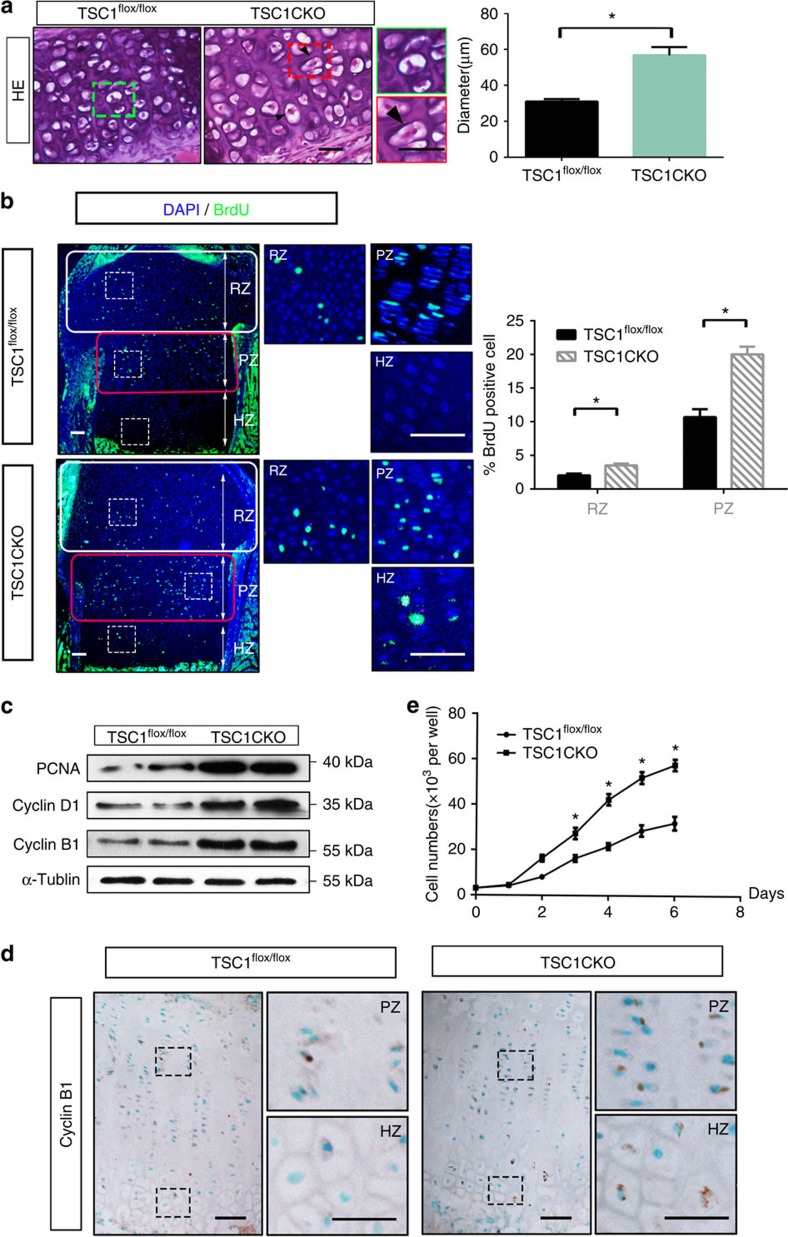
Activation of mTORC1 stimulates chondrocyte growth and proliferation in mice. (**a**) HE staining of rib cartilage in 4-week-old TSC1CKO mice and control mice. Boxed areas on the right represent higher magnification. Triangles indicate chondrocytes under mitosis. Quantification of chondrocyte size. Scale bar, 100 μm. (**b**) BrdU labelling of chondrocytes in tibia of P0 TSC1CKO and control mice. Boxed areas on the right represent RZ, PZ and HZ at a higher magnification. Rounded rectangles represent the counted regions in quantification of BrdU-positive cells. Scale bar, 100 μm. Quantification of BrdU labelling in chondrocytes. BrdU-positive cells are counted in white rounded rectangles (RZ) and red rounded rectangles (PZ). Student's *t*-test, *P<0.05. *n*≥3 mice for each genotype; error bars indicate s.d. (**c**) Western blot analysis showing the expression of PCNA, Cyclin D1 and Cyclin B1 in primary cultured chondrocytes isolated from TSC1^flox/flox^ and TSC1CKO mice at P0. (**d**) Immunofluorescence analysis of Cyclin B1 expression in TSC1CKO and control mouse tibia tissue at 3 weeks. Scale bar, 100 μm. (**e**) CCK8 proliferation assay of primary chondrocytes isolated from TSC1^flox/flox^ and TSC1CKO mice. Student's *t*-test, *P<0.05, *n*≥3; Error bars indicate s.d.

To gain insights into how mTORC1 activation delays endochondral bone development, we compared chondrocyte proliferation and differentiation of TSC1CKO mice with that in WT mice. TSC1 deficiency has been shown to promote cell growth and proliferation in several cell types *in vitro* and *in vivo*^24^,^25^. As expected, cell sizes of TSC1-deficient chondrocytes increased significantly, compared with the littermate controls (Fig. 4a). Proliferation of chondrocytes, determined as a percentage of 5-Bromo-2-deoxyUridine (BrdU)-positive chondrocytes (round, columnar and prehypertrophic chondrocytes) in a BrdU labelling assay, was additionally enhanced in TSC1CKO mice to a significant extent compared with that in WT mice (Fig. 4b). Cell division was observed even in the rib chondrocytes of 8-week-old TSC1CKO mice (Fig. 4a). Upregulation of cyclin D1, cyclin B1 and pCNA further confirmed increased chondrocyte proliferation in TSC1CKO mice (Fig. 4c). Visualization of cyclin B1 in sections of tibias also showed the same results (Fig. 4d). Moreover, mTORC1 activation stimulated proliferation in primary cultures of chondrocytes from TSC1CKO and WT mice *in vitro* (Fig. 4e).

**Figure 5:**
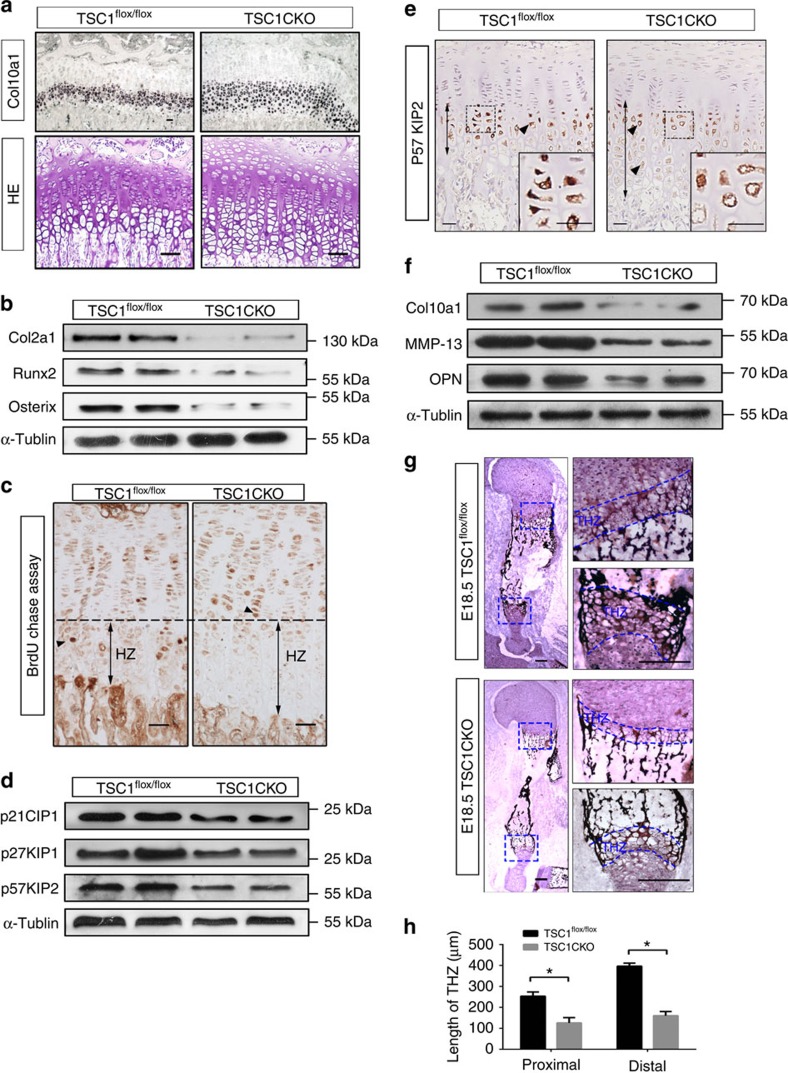
mTORC1 activation inhibits hypertrophic terminal differentiation and ossification. (**a**) *In situ* hybridization analysis of *Col10a1* mRNA and HE staining in TSC1CKO and control mouse tibia at 3 weeks. Scale bar, 100 μm. (**b**) Western blot showing the expression of Col2α1, Runx2 and Osterix in epiphyseal cartilage harvested from 4-week-old TSC1CKO and control mouse tibia. (**c**) BrdU immunohistochemical analysis of tibia from TSC1CKO mouse at 2 weeks after 2 days of BrdU chase assay. The line indicates where differentiation begins. Triangles signify the most distal BrdU-positive chondrocytes. Scale bar, 100 μm. (**d**,**e**) Western blot analysis of p57KIP2, p21CIP1, p27KIP1 (**d**) and immunohistochemical analysis of p57KIP2 (**e**) in tibia of 4-week-old TSC1CKO and control mice. Black triangles show the p57KIP2-positive cells, the arrow lines show the area chondrocytes expressed p57KIP2. Boxed area at the lower right corner represents higher magnification. Scale bar, 100 μm. (**f**) Western blot analysis showing the expression of MMP-13 and OPN in epiphyseal cartilage harvested from 4-week-old TSC1CKO and control mouse tibia. (**g**) Von Kossa staining was performed on the frozen sections of humerus from TSC1CKO and control mice at E18.5. Boxed areas signify terminal hypertrophic chondrocytes undergoing ossification. THZ, terminal hypertrophic chondrocytes zone. Scale bar, 200 μm. (**h**) Graph represents quantification of the width of THZ in TSC1CKO and control mice. Student's *t*-test, *P<0.05, *n*≥3; Error bars indicate s.d.

To investigate the effects of *TSC1* deletion on chondrocyte differentiation in mice, we examined the key markers of chondrocyte identity. *In situ* hybridization analysis of collagen X mRNA revealed marked expansion of collagen X-producing hypertrophic chondrocytes in 3-week-old TSC1CKO tibia (Fig. 5a). Collagen II, Runx2 and Osx levels were markedly decreased in cartilage of TSC1CKO mice (Fig. 5b; Supplementary Fig. 4). *In vivo* BrdU chasing experiments were performed to determine whether accumulation of proliferating chondrocytes was due to decreased rates of chondrocyte hypertrophy. We injected BrdU into 2-week-old mice three times in 6 h to ensure that the majority of proliferative chondrocytes incorporated BrdU, and harvested femurs and tibias 48 h after injection. As only proliferating chondrocytes incorporate BrdU, BrdU-positive chondrocytes in the hypertrophic area were differentiated from proliferative chondrocytes which incorporated BrdU 2 days earlier. BrdU-labelled cells in control mice were distally located in the hypertrophic chondrocyte area while some BrdU-positive cells in TSC1CKO mice remained in the proliferative zone with a typical flat proliferative chondrocyte shape (Fig. 5c). The results indicate that transition of proliferating cells to hypertrophy is delayed due to *TSC1* deletion.

We next determined the underlying reason for significant expansion of hypertrophic chondrocytes in TSClCKO mice. Cyclin-dependent kinase inhibitor 1C (p57KIP2), p21CIP1, p27KIP1, tight binding inhibitors of several G1 cyclin/Cdk complexes and negative regulator of cell proliferation, regulates cell cycle exit and postmitotic hypertrophic differentiation in chondrocytes^26^,^27^,^28^,^29^. We observed downregulation of these proteins, but expansion of p57KIP2 in the hypertrophic zone of TSC1CKO mice was observed, which could explain why TSC1CKO chondrocytes exit the cell cycle and achieve terminal hypertrophy significantly later than their control counterparts (Fig. 5d,e).

Hypertrophic chondrocytes attain terminal stages of differentiation and release the factors responsible for breakdown of matrix and calcification, eventually leading to apoptosis at the chondro-osseous junction. Although chondrocytes expressing Collagen X mRNA was expanded in TSC1CKO mice (Fig. 5a), total Collagen X mRNA and protein level reduced in TSC1CKO tibias, accompanied with an increase of endoplasmic reticulum stress markers GRP78 and XBP1S (Fig. 5f; Supplementary Fig. 5), Other markers of the terminal differentiation stage, OPN and MMP-13, were also markedly downregulated in TSC1CKO mice (Fig. 5f). Von Kossa staining further confirmed that ossification of the growth plate is prevented in TSC1CKO humerus (Fig. 5g,h). HIF1-α, a transcript factor positively regulated by mTORC1 and responsible for chondrocyte survival^16^,^30^,^31^,^32^, was significantly enhanced in TSC1CKO mice (Supplementary Fig. 6). Thus, in the absence of TSC1, chondrocytes failed to develop to the final stages expressing MMP-13 and OPN, subsequently stimulating ossification, despite transition to hypertrophy.

Our results collectively indicate that deletion of *TSC1* stimulates chondrocyte growth and proliferation but prevents hypertrophic and terminal differentiation, leading to disruption of the highly coordinated events of chondrocyte proliferation, differentiation and endochondral ossification, and dwarfness in mice.

### Rapamycin rescues phenotypes in TSC1CKO mice.

**Figure 6:**
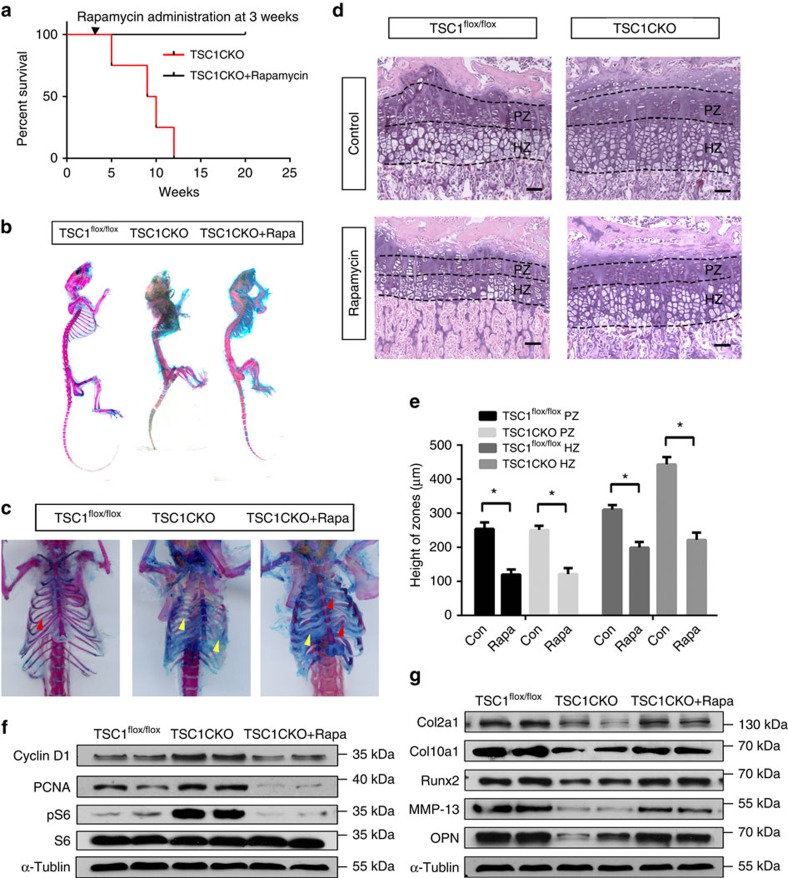
Rapamycin restores normal endochondral bone development. (**a**) Kaplan–Meier Survival curve of TSC1CKO mouse and TSC1CKO mouse treated with rapamycin (2 mg kg^−1^ per day) for 4 weeks at 3 weeks after birth. (**b**,**c**) Alcian blue and Alizarin red staining of skeletons (**b**) and thorax (**c**) at 12 weeks in TSC1^flox/flox^ mouse, TSC1CKO mouse and TSC1CKO mouse administered rapamycin (2 mg kg^−1^ per day). Red triangle shows the ossificated part of the rib, yellow triangle shows the un-ossificated cartilage rib. (**d**,**e**) HE staining (**d**) and quantification analysis (**e**) of 4-week-old control and TSC1CKO mouse tibia treated with or without rapamycin (2 mg kg^−1^ per day) from 2 weeks after birth. Scale bar, 100 μm. Student's *t*-test, *P<0.05, *n*≥3; Error bars indicate s.d. (**f**,**g**) Western blot analysis showing the expression of PCNA, Cyclin D1, pS6 (**f**), and differentiation markers such as Col2a1, Col10a1, RUNX2, MMP-13 and OPN (**g**) in epiphyseal cartilage harvested from TSC1CKO mouse tibia at 4 weeks with or without 2 weeks of rapamycin treatment.

To further ascertain whether defects in endochondronal development are due to activated mTORC1 in TSC1CKO mice, 3-week-old mice were administered the mTORC1-specific inhibitor, rapamycin. After treatment with 2 mg kg^−1^ per day rapamycin for 4 weeks, the lifespan of TSC1CKO mice was extended (Fig. 6a). Alcian blue and Alizarin red staining showed greater bone formation in the rib, leading to a higher volume for lung development (Fig. 6b,c). Accelerated proliferation and expansion of chondrocytes (Fig. 6d,e), expression of cyclin D1 and pCNA were reduced to normal levels (Fig. 6f). Expression levels of collagen II, collagen X, Runx2, MMP-13 and OPN were re-induced in the presence of rapamycin (Fig. 6g), indicating that chondrocytes in TSC1CKO mice re-acquire the ability of hypertrophic differentiation and progress towards terminal hypertrophic differentiation. These phenotypic changes were consistent with the observed dephosphorylation of S6 in rapamycin-treated mice (Fig. 6f). Based on these findings, we propose that mTORC1-dependent chondrodysplasia phenotypes are restored upon inactivation of mTORC1 by rapamycin.

### mTORC1 activity is required for PTHrP transcription

**Figure 7:**
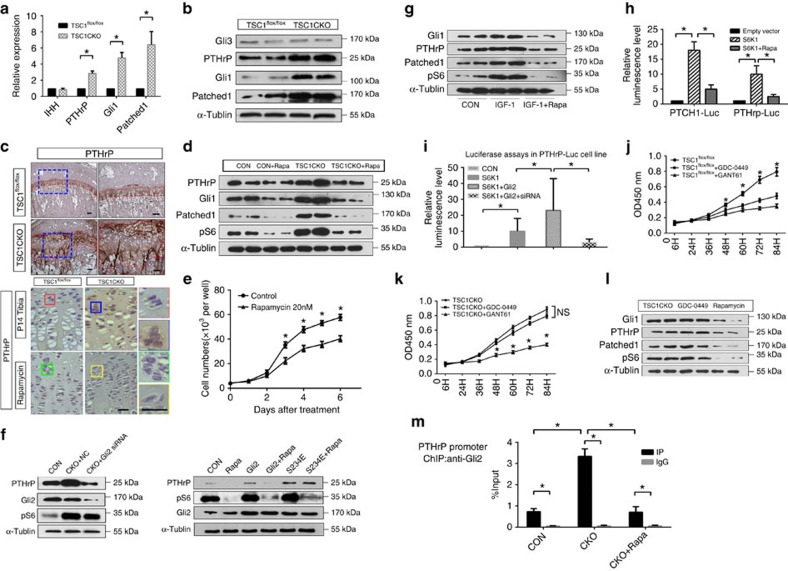
mTORC1 activity is required for PTHrP transcription. (**a**) qPCR analysis of IHH, PTHrP, Patched1, Gli1 in primary cultured chondrocytes isolated from control and TSC1CKO mice. Student's *t*-test, *P<0.05, *n*≥3; Error bars indicate s.d. (**b**) Western blot analysis showing PTHrP, Gli1, Gli3 and Patched1 expression in cartilage tissues from control and TSC1CKO mice. (**c**) Immunohistochemistry analysis showing the expression of PTHrP in 10-week old mice tibia sections. Immunohistochemistry analysis showing the expression of PTHrP in the tibia section of P14 mice which received a week rapamycin injection. Boxed areas are represented by higher magnification on the right. Scale bar, 100 μm. (**d**) Western blot analysis showing the expression of PTHrP, Gli1 and Patched1 in cartilage tissue isolated from control and TSC1CKO mice with rapamycin treated or not. (**e**) Proliferation assays of primary cultured TSC1CKO mouse chondrocytes treated with Rapamycin. Student's *t*-test, NS, not significant, *P<0.05, *n*≥3; Error bars indicate s.d. (**f**) Western blot analysis showing the expression of PTHrp in primary cultured chondrocyte harvested from control and TSC1CKO with Gli2 siRNA, Rapamycin or Gli2 mutant. (**g**) Western blot analysis showing the expression of PTHrP, Gli1 and Patched1 in WT mice primary cultured chondrocytes treated with IGF-1 and rapamycin. (**h**) Luciferase activity in NIH-3T3 cells which were transiently transfected with a S6K1 expression plasmid or control plasmid together with the pGL4.17-PTHrP/ pGL4.17-Patched1 promoter plasmid and pRL-TK plasmid. The Renilla luciferase activities were used as internal controls. One-way analysis of variance (ANOVA) and Dunnett's multiple comparison test, *P<0.05, *n*≥3; Error bars indicate s.d. (**i**) Luciferase assay in PTHrp-Luc cell line transfected with S6K1 or pIRES-S6K1-Gli2 or Gli2 siRNA. One-way ANOVA and Dunnett's multiple comparison test, *P<0.05, *n*≥3; Error bars indicate s.d. (**j**) Proliferation assays of primary cultured control (**j**) and TSC1CKO (**k**) mouse chondrocytes treated with GANT-61, GDC-0449. NS, not significant, One-way ANOVA and Dunnett's multiple comparison test for each time spot, *P<0.05, *n*=5; Error bars indicate s.d. (**l**) Western blot analysis showing the expression of Gli1, Patched1 and PTHrP in primary cultured TSC1CKO mouse chondrocytes treated with rapamycin or GDC-0449. (**m**) CHIP analysis of binding of Gli2 protein to PTHrP gene promoter in choncrocytes treated with Rapamycin or not. Student's *t*-test, *P<0.05, *n*≥3; Error bars indicate s.d.

PTHrP is a main regulator of endochondral bone development under the control of the hedgehog pathway by its downstream effectors in the Gli family. IHH stimulates transcriptional activity of Gli transcription factors toward PTHrP to promote chondrocyte proliferation and inhibit hypertrophic differentiation^16^,^18^,^33^. To gain further insights into the molecular mechanisms through which mTORC1 regulates chondrocyte proliferation and differentiation, we analysed the effects of mTORC1 activation and inhibition on this pathway *in vitro* and in mice. Although the IHH level remained unchanged (Supplementary Fig. 7), markedly higher mRNA and protein levels of the PTHrP and IHH downstream targets, Patched1 and Gli1, were detected in TSC1CKO mice (Fig. 7a,b; Supplementary Fig. 8), indicating that the axis is highly activated by mTORC1. A comparative analysis of TSC1, pS6, PTHrP and Patched expression in growth plate also showed their correlations in chondrocytes of different stages (Supplementary Fig. 9). Consistent with the elevated PTHrP level in TSC1CKO mice and reduced PTHrP level in rapamycin-treated mice (Fig. 7c, the expression of PTHrP targets such as Runx2, p57KIP2 and p27KIP1 was decreased in TSC1CKO mice (Fig. 5b,d). Treatment with rapamycin restored the phenotypes of TSC1CKO mice, since expressions of cyclin D1, PTHrP, Patched1 and Gli1 declined after 2 weeks administration (Fig. 7d). And rapamycin added to primary cultured chondrocytes inhibited proliferation significantly (Fig. 7e). Importantly, we found that mTORC1 activity is crucial for PTHrP expression in chondrocytes under physiological conditions, as evidenced by diminished PTHrP, Patched1 and Gli1 expression in rapamycin-treated WT mice and chondrocytes (Fig. 7d). Downregulation of Gli2 by siRNAs abrogated mTORC1 hyperactivation-stimulated PTHrP expression in TSC1 null chondrocytes, while overexpression of active Gli2 (S234E) counteracted mTORC1 inactivation-reduced PTHrP expression in rapamycin-treated cells (Fig. 7f). Insulin-like growth factor 1 (IGF-1), a physiological chondrogenic factor that is known to stimulate mTORC1, enhanced pS6, Gli1, Patched 1 and PTHrP in an rapamycin-sensitive manner (Fig. 7g).

To further determine whether mTORC1 controlled PTHrP expression by enhancing the transcription of PTHrP via Gli2, luciferase reporter plasmids containing a 2 kb PTHrP promoter or a Patched1 promoter were constructed. We found that rapamycin inhibited, while S6K1 (downstream kinase of mTORC1) overexpression increased PTHrP promoter activity, similar changes were observed on Patched1 promoter activity (Fig. 7h). Further results from luciferase assays showed that siRNA of Gli2 diminished the enhanced luminescence produced by S6K1 overexpression, ruled out the possibility of global effects of mTORC1 signalling on protein synthesis (Fig. 7i). Moreover, although both a Gli1/2 inhibitor, GANT-61 and a IHH receptor smoothened (SMO) antagonist, GDC-0449, repressed proliferation of primary cultured chondrocytes from WT mice, only the Gli1/2 inhibitor, but not the SMO agonist, inhibited the proliferation of chondrocytes from TSC1CKO mice (Fig. 7j,k). Accordingly, rapamycin, but not GDC-0449 reduced Gli1, Patched1 and PTHrP expression in TSC1 null chondrocytes (Fig. 7l). Importantly, chromatin immunoprecipitation (ChIP) assay further confirmed the mTORC1-dependent binding of Gli2 to *PTHrP* gene promoter in choncrocytes (Fig. 7m). These results imply that mTORC1 stimulates hedgehog/PTHrP signalling in chondrocytes through Gli1/2 in an IHH receptor-independent manner.

### mTORC1/S6K1 regulates PTHrP transcription through Gli2

**Figure 8:**
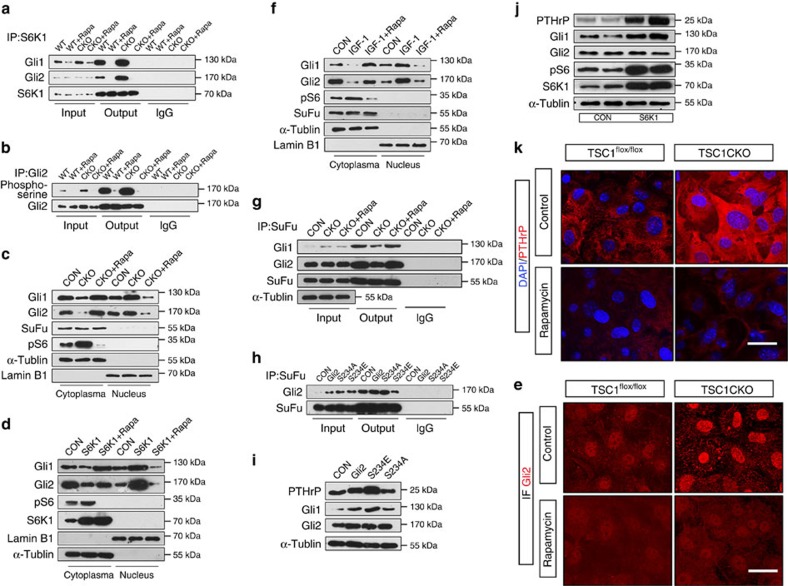
mTORC1/S6K1 activates Gli2 to promote PTHrP transcription. (**a**) Chondrocytes from control and TSC1CKO mice with or without rapamycin treatment were lysed and the interactions between S6K1 and Gli2 or Gli1 were analysed by IP-western analysis. (**b**) IP-western analysis showing the phosphorylation of Gli2 in primary cultured chondrocytes with or without rapamycin treatment. (**c**) Analysis of subcellular fractionation of Gli2 and Gli1 in cultured chondrocytes from control and TSC1CKO mice. (**d**) Analysis of subcellular fractionation of Gli2 and Gli1 in normal ATDC5 cells and ATDC5 cells transfected with the S6K1 expressing plasmid. (**e**) Immunofluorescence of Gli2 in cultured chondrocytes from control and TSC1CKO mice with or without rapamycin treatment showed the translocation of Gli2. Scale bar, 25 μm. (**f**) Analysis of subcellular fractionation of Gli2 and Gli1 in normal ATDC5 treated with IGF-1 and rapamycin. (**g**) Immunoprecipitation analysis showing the interactions of SuFu and Gli1/2, SuFu and S6K1 in normal chondrocytes or TSC1 null chondrocytes treated with rapamycin or not. (**h**) Immunoprecipitation analysis showing the interactions of SuFu, Gli2 and its mutants S234A, S234E. (**i**) Western analysis showing the expression of Gli1, PTHrP, Gli2 in ATDC5 cells transiently transfected with Gli2 and its mutants. (**j**) Western analysis showing the expression of PTHrP, Gli1, Gli2, pS6 and S6K1 in ATDC5 cells and ATDC5 cells transiently transfected with S6K1 expression vector. (**k**) Immunofluorescence of PTHrP in cultured chondrocytes from control and TSC1CKO mice with rapamycin treatment. Scale bar, 25 μm.

The potential mechanisms by which mTORC1 activates the hedgehog/PTHrP axis without affecting IHH expression were further explored. A recent study has shown that the mTORC1 substrate, S6K1, directly regulates hedgehog signalling in cancer cells by interacting with and phosphorylating Gli1, but not Gli2 via a SMO-independent mechanism^34^. Consistent with this report, we confirmed the interaction between S6K1 and Gli1 in chondrocytes (Fig. 8a). Interestingly, we found that S6K1 also interacted with Gli2 (Fig. 8a), a main regulator of PTHrP in chondrocytes^35^,^36^,^37^, without forming a Gli1 and Gli2 dimeric complex in the cells (Supplementary Fig. 10). In contrast, Gli3 was not associated with S6K1 in chondrocytes (Supplementary Fig. 10). Inhibition of mTORC1 by rapamycin treatment suppressed the interactions of S6K1 with Gli1/2 in chondrocytes (Fig. 8a). In contrast, mTORC1 activation enhanced the interactions of S6K1 with Gli1/2 in chondrocytes from TSC1CKO mice (Fig. 8a). Also, rapamycin reduced these interactions in TSC1CKO chondrocytes (Fig. 8a). Importantly, mTORC1 inhibition reduced, while mTORC1 activation enhanced serine phosphorylation of Gli2 (Fig. 8b).

Activation of the hedgehog pathway promotes the dissociation of cytoplasmic Sufu-Gli complexes allowing the subsequent nuclear translocation of Gli proteins to activate Gli-dependent transcription^38^, and the recognizing motif of S6K1 is near the SuFu binding site in Gli2 (ref. 39). Thus, we speculate that phosphorylation of Gli2 may release Gli2 from its endogenous inhibitor SuFu. To investigate whether S6K1 affects the localization of endogenous Gli2 by inhibiting SuFu binding, we examined Gli2 expression in cytoplasm and nucleus in TSC1CKO chondrocytes and ATDC5 cells which were transiently transfected with S6K1 expression plasmid. The results showed that more Gli2 in these cells was localized in the nucleus fraction, especially significant in S6K1 transfected cells (Fig. 8c,d). We also visualized Gli2 by immunofluorescence in chondrocytes and found that loss of TSC1 resulted in more nucleus translocation of Gli2 and rapamycin treatment inhibited this process (Fig. 8e). Importantly, IGF-1, also enhanced the nuclear translocation of Gli1/2 in an rapamycin-sensitive manner (Fig. 8f).

Accordingly, the interaction between SuFu and Gli2 was markedly reduced in TSC1 null cells, while rapamycin treatment restored the interaction, suggesting that activated S6K1 may inhibit SuFu binding with Gli2 (Fig. 8g). Consistent with this result, no SuFu protein was detected after immunoprecipitation by S6K1 antibody (Supplementary Fig. 11). Furthermore, mutation in the S6K1 phosphorylation site on Gli2 (S234A) blocked the ability of S6K1 to break the interaction between Gli2 and Sufu (Fig. 8h) and the effect of mTORC1 on Gli1, Gli2 and PTHrP expression (Fig. 8i). On the contrary, the active mutant of Gli2(S234E) showed opposite effects on the interaction and Gli1, Gli2 and PTHrP expression(Fig. 8h-i). These findings demonstrated that phosphorylation of Gli2 by S6K1 releases Gli2 from SuFu, resulting in the translocation of Gli2 from cytoplasm to nucleus.

To further determine whether S6K1-induced Gli2 nuclear translocation stimulates PTHrP expression, we transfected ATDC5 cells with S6K1 expression vector and the results showed that these cells expressed much more PTHrP than the control cells (Fig. 8j), which is consistent with the results in TSC1CKO cells. This result was confirmed by immunofluorescence of PTHrP in chondrocytes (Fig. 8k). Similar results were observed in TSC1CKO chondrocytes by immunohistochemistry (Fig. 7c).

Taken together, these findings suggest that mTORC1/S6K1 regulates PTHrP transcription by phosphorylating and promoting Gli2 nuclear accumulation via inhibition of SuFu binding.

## Discussion

**Figure 9:**
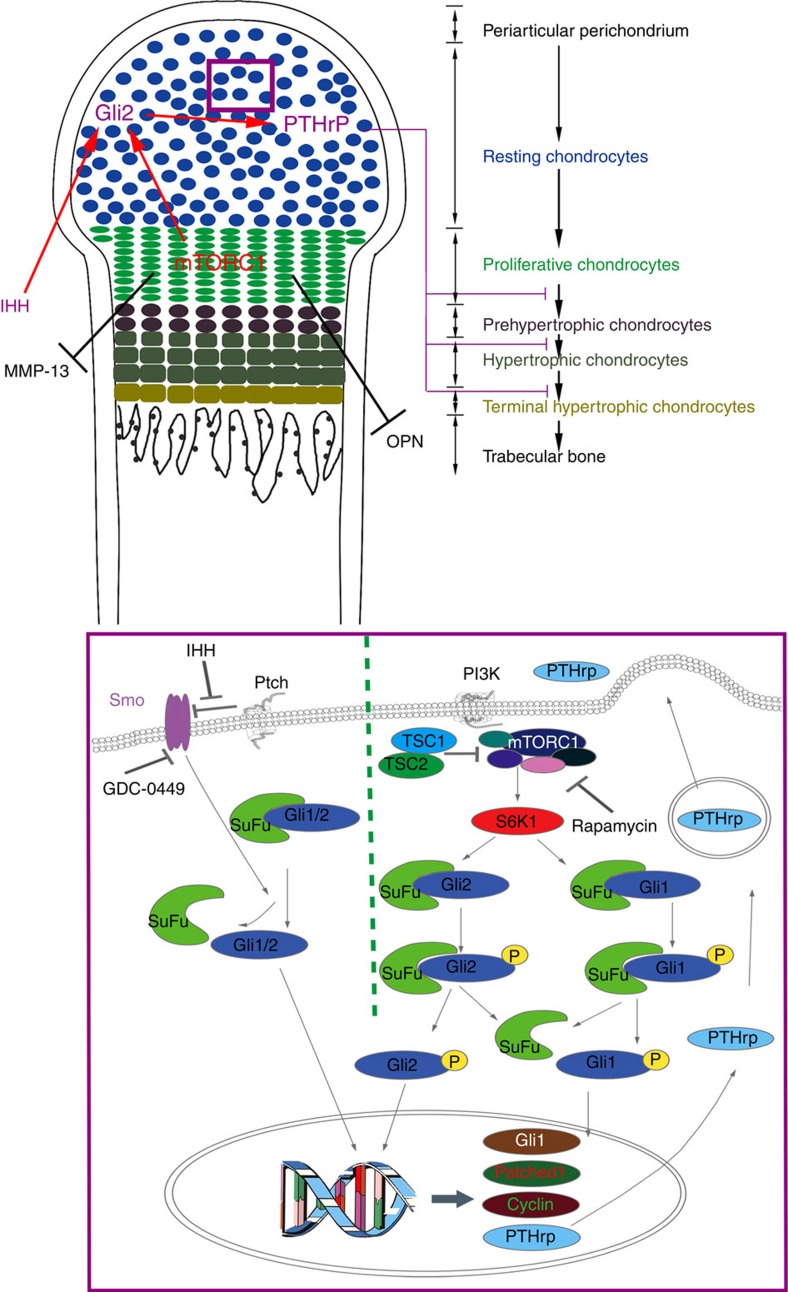
mTORC1/S6K1 participation in the functional production of PTHrP. A schematic model depicting the role of mTORC1 in the regulation of PTHrP and coordination of chondrocyte proliferation and differentiation during endochondral bone development.

We have established that precise regulation of mTORC1 activity is crucial for coordination of chondrocyte proliferation and differentiation during endochondral skeleton development. We demonstrate that mTORC1 activity is required for chondrocyte growth and proliferation, but declines during the process of differentiation *in vitro* and *in vivo*. Low mTORC1 activity is necessary for chondrocyte differentiation and maturation. Hyperactivation of mTORC1 in chondrocytes uncouples the normal proliferation and differentiation programme within the growth plate, leading to the impairment of chondrocyte hypertrophic and terminal differentiation, chondrodysplasia and dwarfness in mice. Mechanistically, mTORC1 downstream kinase S6K1 interacts with and phosphorylates transcription factor Gli1/2, promotes its nuclear accumulation and initiates PTHrP transcription, subsequently promotes chondrocyte proliferation and prevents chondrocyte hypertrophic and terminal differentiation (Fig. 9). Thus, we established the vital role of the mTORC1 pathway in controlling PTHrP expression during endochondral skeleton development.

Rapamycin has been shown to suppress chondrocyte proliferation and endochondral bone growth *in vitro*^23^,^40^, and in chicken embryo^21^ and growing young rats^41^. Recent experiments demonstrated that deletion of either mTOR or Raptor in mouse limb bud mesenchyme using Prx1-Cre leads to diminished embryonic skeletal growth due to reduction in chondrocyte cell size and the amount of cartilage matrix, and subsequent severe delay in chondrocyte hypertrophy and bone formation^20^. Here, we established that constitutive activation of mTORC1 in chondrocytes increases cell size and enhances proliferation *in vitro* and *in vivo*. These experiments collectively suggest a critical requirement of mTORC1 for chondrocyte growth and proliferation.

Limited reports are available on the role of mTORC1 in chondrocyte differentiation. Inhibition of mTORC1 was previously shown to prevent chondrocyte differentiation *in vivo* and *in vitro*^23^,^40^,^41^, implying a positive role in chondrocyte differentiation and maturation. Disruption of either phosphatase tensin homologue (PTEN) or liver kinase b1 (Lkb1) using Col2a1-Cre, both leading to activation of mTORC1 in chondrocytes, unexpectedly produced opposite phenotypes. PTEN-deleted mice exhibited accelerated hypertrophic differentiation, matrix overproduction and increased skeletal size^42^. In contrast, loss of Lkb1 led to diminished transition of cells to hypertrophy, dramatic expansion of mitotic chondrocytes, and formation of enchondroma-like tumours throughout long bones^43^. However, mice with *TSC1* disruption in this study demonstrated impairment of hypertrophic chondrocytes and blockage of terminal differentiation of hypertrophic chondrocytes, delayed ossification and dwarfness phenotypes. These distinct phenotypes may be attributed to the activation of mTORC1-independent pathways in *PTEN* or *Lkb1* deletion mice. Akt is highly activated in *PTEN*, but not *Lkb1* or *TSC1* deletion mice. AMP-activated protein kinase, the major target of Lkb1, is known to regulate several signalling pathways other than mTORC1 (refs 44, 45). Tumour formation has been reported in *Lkb1*, but not *TSC1* or *PTEN* deletion mice, further suggesting that mTORC1-independent mechanisms contribute to the phenotypes of *Lkb1* null mice. In contrast to PTEN and Lkb1, mTORC1 is a well-established exclusive target of TSC1/2 (ref. 46). Rapamycin reversed phenotypes in TSC1CKO mice in our experiments, confirming that the effects of *TSC1* deletion are mTORC1 dependent. Prevention of chondrocyte differentiation upon mTORC1 inhibition in previous studies could be explained by the fact that obtaining the appropriate cell size and number during growth and proliferation is necessary before differentiation^20^,^22^,^24^. Impaired differentiation may be a consequence of inhibition of cell growth and proliferation by rapamycin. This theory was supported by our finding that rapamycin treatment at later stages promotes chondrocyte differentiation but inhibits this process at early stages *in vitro*. Importantly, mTORC1 activity declined during the normal chondrocyte differentiation process *in vitro* and in developing long bone. Clearly, low mTORC1 activity is essential for chondrocyte differentiation, and its hyperactivation prevents differentiation and endochondral bone growth.

IHH and PTHrP coordinate chondrocyte proliferation and maturation through a negative feedback mechanism. IHH produced by pre- and early hypertrophic chondrocytes stimulates chondrocyte proliferation and regulates PTHrP, Patched1, collagen X and Gli1 expression by activating the transcription factor Gli2. PTHrP, in turn, suppresses chondrocyte maturation associated with IHH expression^12^,^14^,^16^. Various studies have reported distinct effects of rapamycin on IHH expression (increase, decrease or no effect)^21^,^40^,^41^. Although we observed no significant changes in IHH expression, its downstream targets, Patched1, Gli1 and PTHrP, were markedly enhanced upon mTORC1 activation (Fig. 7a,b; Supplementary Fig. 7,8). Key hypertrophic regulators such as Runx2, p57KIP2 and p27KIP1, which are negatively regulated by PTHrP, were decreased in TSC1CKO mice. Our further studies revealed that mTORC1 may regulate hedgehog/PTHrP signalling to prevent chondrocyte differentiation by stimulating Gli2 in an IHH receptor-independent mechanism. Nevertheless, PTHrP-independent deregulation of the hypertrophic differentiation pathway might also contribute to the phenotypes in TSC1CKO mice.

It has been well established that Gli2 plays a vital role in regulating PTHrP expression in chondrocytes^36^,^37^. Recent studies reported that S6K1 interacts with Gli1, but not Gli2 in cancer cells^34^,^47^. Similarly, in chondrocytes, we found that S6K1 also interacted with Gli2 in addition to Gli1. This finding is not surprising, as both Gli1 and Gli2 contain a similar S6K1 recognizing motif, RKRALS^39^. Upon mTORC1 activation, Gli2 could be phosphorylated by S6K1 and released from the binding of its endogenous inhibitor SuFu, allowing it to translocate into the nucleus to initiate transcription. Significant nucleus localization of Gli2 was observed in TSC1CKO chondrocytes and S6K1 overexpressing cells. Importantly, rapamycin reduces S6K1-Gli2 interaction and PTHrP expression in normal chondrocytes, indicating an essential role of mTORC1 in PTHrP transcription. A recent study by Chen *et al*.^20^ demonstrates that deletion of mTORC1 (Raptor) has a global decrease of protein synthesis in chondrocytes. Although the possible involvement of global effects of mTORC1 signalling on protein synthesis could not be excluded, our results strongly support that mTORC1/S6K1 is required for PTHrP transcription by phosphorylating and promoting Gli2 nuclear accumulation via inhibition of SuFu binding.

In conclusion, normal and precisely controlled mTORC1 activity is essential to coordinate chondrocyte growth, proliferation and differentiation during endochondral bone formation, and our data have established a novel link between the nutrient-sensing pathway and chondrocyte proliferation and differentiation, and identified a novel mechanism for regulation of PTHrP expression by mTORC1/S6K1. Alterations in mTORC1 activity are sufficient to induce a switch from chondrocyte differentiation to proliferation. Further studies are warranted to elucidate how mTORC1 activity is regulated during endochondral bone development under physiological or pathological conditions.

## Methods

### Mouse husbandry and genotyping

Animal experiments were approved by the Ethical Committee for Animal Research of Southern Medical University and conducted based on the state guidelines from the Ministry of Science and Technology of China. All C57BL/6 mice (newborn or 4–5 weeks old, Mice were used regardless of gender.) were purchased from the Laboratory Animal Center of Southern Medical University. TSC1 floxed mice were purchased from the Jackson Laboratory. The Col2a1-cre mouse line was a generous gift from Dr Xiao Yang (Academy of Military Medical Sciences, Beijing, PRC). To generate chondrocyte-specific TSC1 deletion mice, TSC1 floxed mice line were crossed with Col2a1-cre mice. Mice at different ages were used regardless of gender. The primers involved in the genotyping of mice are listed in Supplementary Table 1.

*Antibodies*. The following antibodies were used: rabbit anti-pS6 (S235/236) (1:1,000 for WB, 1:100 for IHC/IF, CST, clone D57.2.2E, 4858), mouse anti-S6(1:3,000 for WB, Santa Cruz, clone C-8, sc-74459), rabbit anti-pAKT(S473) (1:1,000 for WB, CST, clone D9E, 4060), rabbit anti-AKT (1:1,000 for WB, CST, clone C67E7, 4691), mouse anti-Col II (1:1,000 for WB, 1:200 for IHC/IF, Abcam, clone 5B2.5, ab3092), rabbit anti-ColX (1:1,000 for WB, Abcam, ab58632), rabbit anti-IHH (1:200 for IHC/IF, Bioworld, BS8306), rabbit anti-PTHrP (1:500 for WB, 1:20 for IHC/IF, Santa Cruz, sc-20728), rabbit anti-PCNA (1:500 for WB, Bioworld, BS5842), mouse anti-BrdU (1:1,000 for IHC/IF, Sigma, clone BU-33, b8434), rabbit anti-Cyclin D1 (1:500 for WB, CST, clone 92G2, 2978), rabbit anti-Cyclin B1 (1:500 for WB, 1:50 for IHC/IF, Boster, pb0130), rabbit anti-Gli1 (1:2,000 for WB, 1:100 for IP, Santa Cruz, sc-20687X), rabbit anti-Gli2 (1:1,000 for WB, 1:100 for IHC/IF, Abcam, ab167389), rabbit anti-Gli2 (1:2,000 for WB, 1:1,000 for IP/CHIP, Santa Cruz, sc-28674X), rabbit anti-Gli3 (1:500 for WB, Proteintech, 19949-1-AP), rabbit anti-Patched1 (1:500 for WB, Proteintech, 17520-1-AP), rabbit anti-Patched1 (1:500 for WB, 1:50 for IF, Santa Cruz, sc-6149), rabbit anti-SuFu (1:1,000 for WB, 1:100 for IP, CST, clone C81H7, 2522), rabbit anti-Osterix (1:1,000 for WB, 1:100 for IHC/IF, Abcam, ab22552), rabbit anti-P57KIP2 (1:500 for WB, 1:100 for IHC/IF, Bioworld, BS6876), rabbit anti-MMP-13 (1:1,000 for WB, Abcam, ab39012), rabbit anti-S6K1 (1:1,000 for WB, 1:100 for IHC/IF, 1:100 for IP, Santa Cruz, sc-9027), rabbit anti-GRP78 (1:1,000 for WB, 1:50 for IHC/IF, Bioworld, BS6479), rabbit anti-XBP1S (1:400 for WB, Santa Cruz, sc-7160), rabbit anti-TSC1 (1:1,000 for WB, 1:100 for IHC/IF, Santa Cruz, sc-13013), rabbit anti-OPN (1:500 for WB, Abcam, ab8448), rabbit anti-Runx2 (1:1,000 for WB, 1:200 for IHC/IF, Abcam, ab23981), rabbit anti-P27KIP1 (1:1,000 for WB, CST, clone D37H1, 3688), rabbit anti-P21CIP1 (1:1,000 for WB, CST, clone 12D1, 2947), mouse anti-α-Tublin (1:3,000 for WB, Rayantibody, clone MG17, RM2007), mouse anti-GAPDH (1:6,000 for WB, Rayantibody, clone MC4, RM2002), rabbit anti-Lamin B1 (1:1,000 for WB, CST, clone D9V6H, 13435), Goat Anti-Rabbit IgG Light Chain (1:1,000 for WB, Abbkine, A25022), mouse Anti-Phospho-Thr/Ser (1:1,000 for WB, Raybiotech, RM3008) and rabbit anti-Phospho-Serine (1:2,000 for WB, Immunechem, ICP9806), HRP labelled Goat Anti-Rabbit IgG (H+L) (1:1,000 for WB, 1:100 for IHC, Jackson ImmunoResearch, 111-035-003), HRP labelled Goat Anti-Mouse IgG (H+L) (1:3,000 for WB, Jackson ImmunoResearch, 115-035-003), Alexa Fluor 594 labelled Goat Anti-Rabbit IgG H&L (1:500 for IF, Abcam, ab150080), Alexa Fluor 488 labelled Goat Anti-Rabbit IgG H&L (1:500 for IF, Abcam, ab150077), Alexa Fluor 488 labelled Goat Anti-Mouse IgG H&L (1:500 for IF, Abcam, 150113).

*Enzymes*. Taq mix for genotyping (DreamTaq PCR Master Mix, Thermo), High Fidelity PCR enzyme (KOD-Plus-Neo, Toyobo), DpnI (Thermo), EcoRI (Thermo), XbaI (Thermo) and T4 DNA ligase (Thermo).

*siRNA*. siRNA of Gli2 was purchased from Santa Cruz (sc-37913).

### Cell lines and chondrocyte primary culture

NIH-3T3 Cell line was purchased from ATCC (Rockville, USA) and grown in DMEM medium (Gibco, USA) supplemented with 10% fetal bovine serum (Life Technologies, USA). ATDC5 cell line was supplied by Riken BioResource Center (Tsukuba, Japan), and maintained at 37 °C under 5% CO_2_ in DMEM/F12 medium (Gibco, USA) supplemented with 5% fetal bovine serum (Life Technologies, USA). Chondrocytes were obtained from rib cartilage of newborn mice. Rib cartilage was dissected from several mouse pups under a stereo light microscope. After digestion in 0.1% collagenase type II (Sigma) for 4 h, chondrocytes were seeded in a 12-well plate at a density of 5 × 10^3^ cells per ml and cultured at 37 °C with 5% CO_2_. DMEM/F12 with L-glutamine (Gibco) supplemented with 1% penicillin-streptomycin solution (Life Technologies) and 10% fetal bovine serum (Gibco) was used as the culture medium. ITS-inducing culture was initiated at P2 by adding 10 mg l^−1^ insulin (Sigma), 5.5 mg l^−1^ human transferrin (Sigma), 3 × 10^−8^ mol l^−1^ sodium selenite (Sigma) and 1 nM bone morphogenetic protein-2 into the chondrocyte primary culture medium.

### Inhibitor treatment

For the proliferation assays, cells were seeded in 96-well plates at the density of 5 × 10^3^ cells per well. Cells were treated with or without 10 μM GDC-0449 (Selleck), 5 μM GANT-61 (Selleck), 10 nM rapamycin (Sigma) and OD450 nm values were obtained every 12 h. Rapamycin (1.5 or 2 mg kg^−1^ per day) were administered to 3-week-old mice for 1–4 weeks before sacrifice. Mice under 3 weeks were received 0.75 mg kg^−1^ per day rapamycin by intraperitoneal injection.

### Immunofluorescence staining and immunohistochemistry

Bones of mice harvested at different ages were fixed in 4% paraformaldehyde (PFA) overnight at 4 °C and decalcified in 0.5 M EDTA, pH 7.4, on a shaker for 1–3 weeks. Bones were either embedded in paraffin or stored at −80 °C in Optimal Cutting Temperature Compound (OCT). Immunofluorescence staining was performed on 5 μm frozen sections or 2 μm paraffin sections. Immunohistochemical analysis was conducted on 2 μm paraffin sections. After deparaffinization and rehydration, sections were incubated in citrate buffer (10 mM citric acid, pH 6.0) for 20 min at 90 °C or treated with 200 μg ml^−1^ proteinase K (Sigma) for 10 min at 37 °C to unmask antigen. Sections for IHC were treated with 3% hydrogen peroxide for 10 min. After that, the sections were permeabilized with 0.1% Triton X-100 in PBS for 5 min at room temperature and then blocked with 1% sheep serum at room temperature for 1 h. Then sections were immunostained with primary antibodies (in 1% BSA, 0.1% Triton X-100) at 4 °C overnight. For secondary reactions, species-matched Alexa Fluor 488-, Alexa Fluor 594- or HRP-labelled secondary antibody was used (1:500 in 1% BSA, 1 h) at 37 °C. For IHC, DAB was used as chromogen, hematoxylin was used to counterstain. For IF, sections were mounted with DAPI (Thermo) before imaging. Skeletal staining was performed on skeletons of 12-weeks mice with Alcian blue (Sigma) and Alizarin red (Sigma).

### Plasmid construction and *in situ* hybridization

Promoters of *PTHrP* and *Patched1* were cloned into pGL4.17 plasmid (Promega) separately. A fragment of *Col10a1, TSC1, MMP-13* mRNA were cloned into a pBSK+ plasmid separately. Digoxigenin-labelled cRNA probe was produced by T3 RNA Polymerase and in hybridization was performed using anti-Digoxigenin-AP (Roche), NBT/BCIP was used as chromogen^48^,^49^. The CDS of human Gli2 were amplified from pCS2-MT GLI2 FL, then cloned into the plasmid pCDNA3.1(+). pCS2-MT GLI2 FL was a gift from Erich Roessler (Addgene plasmid #17648). Gli2 mutants S234A, S234E were constructed by point mutation from pCDNA3.1(+)-Gli2. The plasmid expressed S6K1 and Gli2 simultaneously was pIRES-S6K1-Gli2. All primers are listed in Supplementary Table 1.

### Cell proliferation assay

Cell proliferation was determined using a modified 3-(4,5-dimethyl-2-thiazolyl)-2,5 -diphenyl-2H-tetrazolium bromide assay employing the Cell Counting kit 8 (CCK8; Dojindo) according to the manufacturer's protocol. The data were collected from three independents experiments.

### Immunoblotting and co-immunoprecipitation

Immunoblotting was performed as described in our previous studies^50^. To lyse cartilage, tissue was frozen and ground into a powder using liquid nitrogen in a mortar. For co-immunoprecipitation, 2 × IP lysis buffer (IP buffer: Tris-HCl (pH7.5) 50 mM, NaCl 150 mM, EDTA-2Na 1 mM, Triton X-100 1%, Na_4_P_2_O_7_·10H_2_O 1 mM, Na_3_VO_4_·H_2_O 1 mM) was used to lyse tissue powder or cultured cells. Lysates were incubated with anti-S6K1 antibody (Santa Cruz) or anti-SuFu antibody (CST) overnight at 4 °C under gentle rotation. After protein A/G-agarose beads were added, the mixtures were gently rotated for 2 h at 4 °C. The beads were collected by centrifugation at 6,000 r.p.m. and gently washed three times with immunoprecipitation buffer. The beads were lysed with 2 × SDS–polyacrylamide gel electrophoresis sample buffer and standard immunoblottings were performed with different antibodies. Uncropped western blots scans are provided in the Supplementary Fig. 12a-h.

### ChIP assay

Chromatin from cultured cells was fragmented by using the EZ-Zyme Chromatin Prep Kit (Millipore), and ChIP assay was performed according to the manufacturer's instructions. Primers that were used in the ChIP assay are listed in Supplementary Table 1.

### PCR and qRT–PCR

Genotyping was conducted by PCR with the tissue samples from mice. Detection of deleted allele was performed on DNA samples extracted from various tissues from TSC1CKO mice. Data is presented in Supplementary Fig. 2, original picture is provided in Supplementary Fig. 12i.

Total RNA was extracted from tissues or cultured cells and cDNA synthesized with the TaKaRa PrimeScript RT reagent Kit. qRT–PCR was performed according to the instructions of TaKaRa SYBR Premix Ex Taq. The primers used are described in Supplementary Table 1. Dissociation curves were obtained to ensure the specificity of each qRT–PCR reaction. GAPDH was used as the internal control for normalization.

### Dual-luciferase reporter assay

NIH-3T3 cells were seeded into six-well plates 1 day before transfection. Cells were co-transfected with 1,800 ng of pGL4.17 DNA or pGL4.17-PTHrP-promoter/pGL4.17-Patched1 promoter, together with 900 ng pCDNA3.1-Flag-S6K1 and 200 ng of pRL-TK DNA (Renilla luciferase, Promega) with lipofectamine 2000 transfection reagent (Life Technologies). The PTHrp promoter cell line was established by introducing pGL4.17-PTHrP-promoter plasmid into NIH-3T3 cells. And a stable PTHrp-luc cell line was obtained after 2 week selection by 600 μg ml^−1^ G418 (Clontech). Forty-eight hours after transfection, luciferase activity was analysed using a dual-luciferase assay kit (Promega) according to the manufacturer's protocol. Firefly luciferase activity was normalized to Renilla luciferase activity.

### BrdU incorporation assay

For BrdU incorporation, newborn mice were administered a single intraperitoneal injection of 1 ml BrdU (Invitrogen) per 100 g body weight. Animals were sacrificed 2 h after injection. In the BrdU chase assay, 2-week-old mice received two pulses of BrdU with an intervening interval of 6 h, and sacrificed 48 h after the first injection, to ensure that BrdU-labelled chondrocytes have enough time to differentiate^51^. Long bones were harvested, fixed in 4% PFA, decalcified in EDTA, and embedded in paraffin. Visualization of BrdU was performed via immunohistochemistry or immunofluorescence with the anti-BrdU antibody (Sigma).

### Statistical analysis

All experiments were performed in triplicate. Data are presented as mean±s.d. unless otherwise indicated. SPSS 13.0 software was used for statistical analyses. Differences between groups were analysed using Student's *t*-test, One-way analysis of variance and Dunnett's multiple comparison test. And a level of *P*<0.05 was considered statistically significant.

## Additional information

**How to cite this article:** Yan, B. *et al*. mTORC1 regulates PTHrP to coordinate chondrocyte growth, proliferation and differentiation. *Nat. Commun.* 7:11151 doi: 10.1038/ncomms11151 (2016).

## Supplementary Material

Supplementary Information Supplementary Figures 1-12 and Supplementary Table 1.
