# Supplementary material for: mTORC1 regulates PTHrP to coordinate chondrocyte growth, proliferation and differentiation
Source: Nat Commun. 2016 Apr 4;7:11151. doi: 10.1038/ncomms11151 (PMC4822018; doi:10.1038/ncomms11151)
Supplement: Supplementary Information — Supplementary Figures 1-12 and Supplementary Table 1. [file ncomms11151-s1.pdf]

SUPPLEMENTARY INFORMATION

Supplementary Figures

Supplementary Figure 1

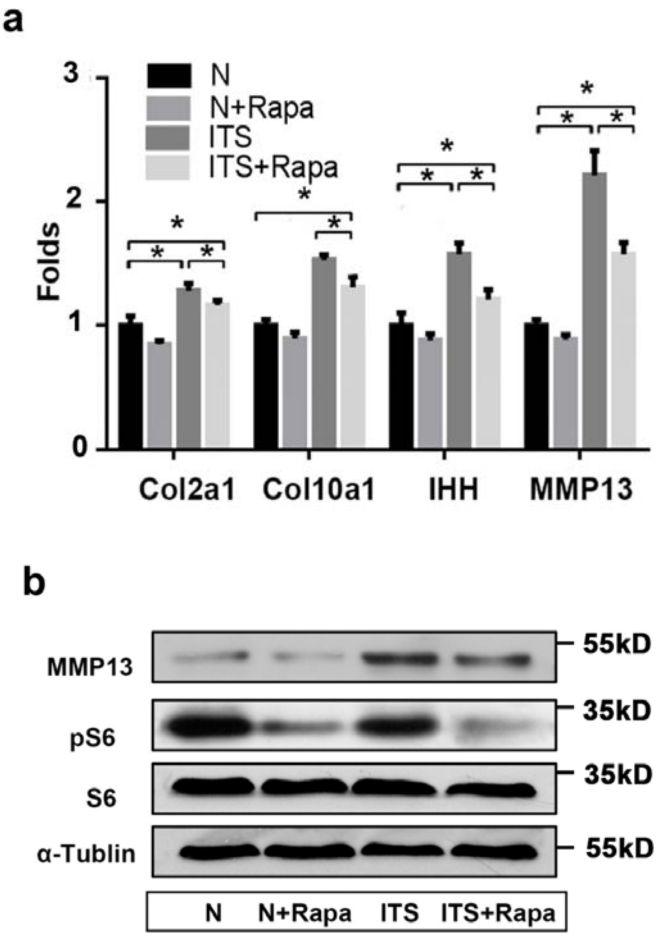

Supplementary Figure 1. Rapamycin prevent early differentiation of chondrocyte. (**a**). qPCR and immunoblotting (**b**) analysis of primary chondrocytes cultured in growth medium or ITS medium. Cells were treated with or without rapamycin (10 nM) from day 1 and were harvested on day 14 after confluence. N, normal medium; ITS, Insulin-transferrin-selenium medium; Rapa, rapamycin. One-way ANOVA and Dunnett's multiple comparison test, *P<0.05, n≥3; Error bars indicate S.D.

Supplementary Figure 2

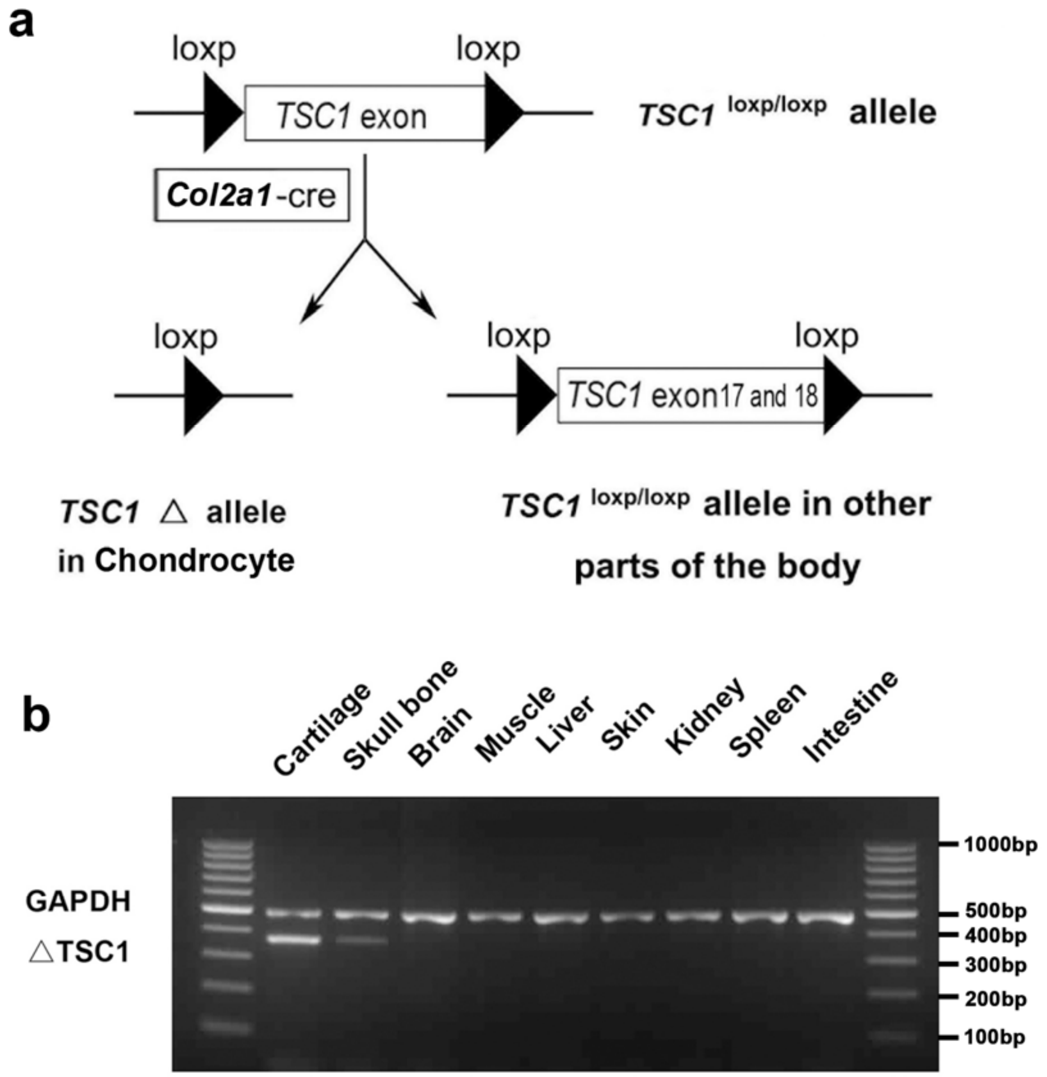

Supplementary Figure 2. Generation of mice with chondrocyte-specific deletion of *TSC1*. (a) Schematic of deletion of *TSC1* by *Col2a1*-cre-mediated recombination. This mutant carries a "floxed" allele of *TSC1,* when combined with a mutant carrying *Col2a1*-Cre recombinase gene, exons 17 and 18 of *Tsc1* are deleted in the chondrocytes. **(b)** Genotyping the offspring after mating transgenic Cre and loxp mice. Deletion of *TS*C1 gene in different tissues were shown by amplification of deleted DNA fragment. Original image of agarose gel electrophoresis is provided in Supplementary Fig. 12.

Supplementary Figure 3

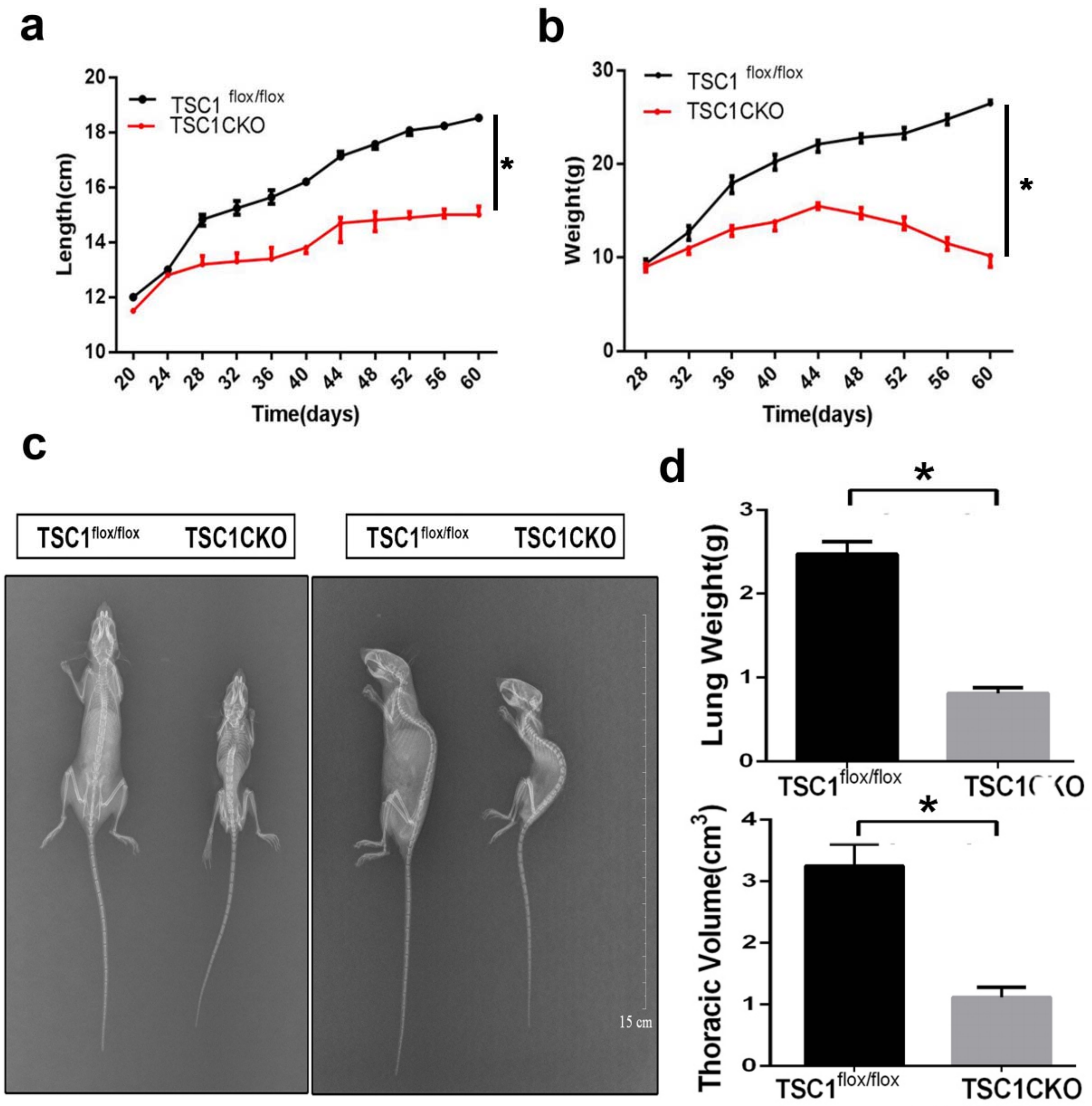

Supplementary Figure 3. General phenotypes of TSC1CKO mice. (**a-b**) The growth curves of control mice and TSC1CKO mice. (**c**) X-ray imaging of TSC1CKO mice at 4 weeks old. Paired sample T test, P<0.05, n=4. (d) Thoracic volume and Lung weight of control mice and TSC1CKO mice at 4 weeks old. Student's t test, P<0.05, n=5.

Supplementary Figure 4

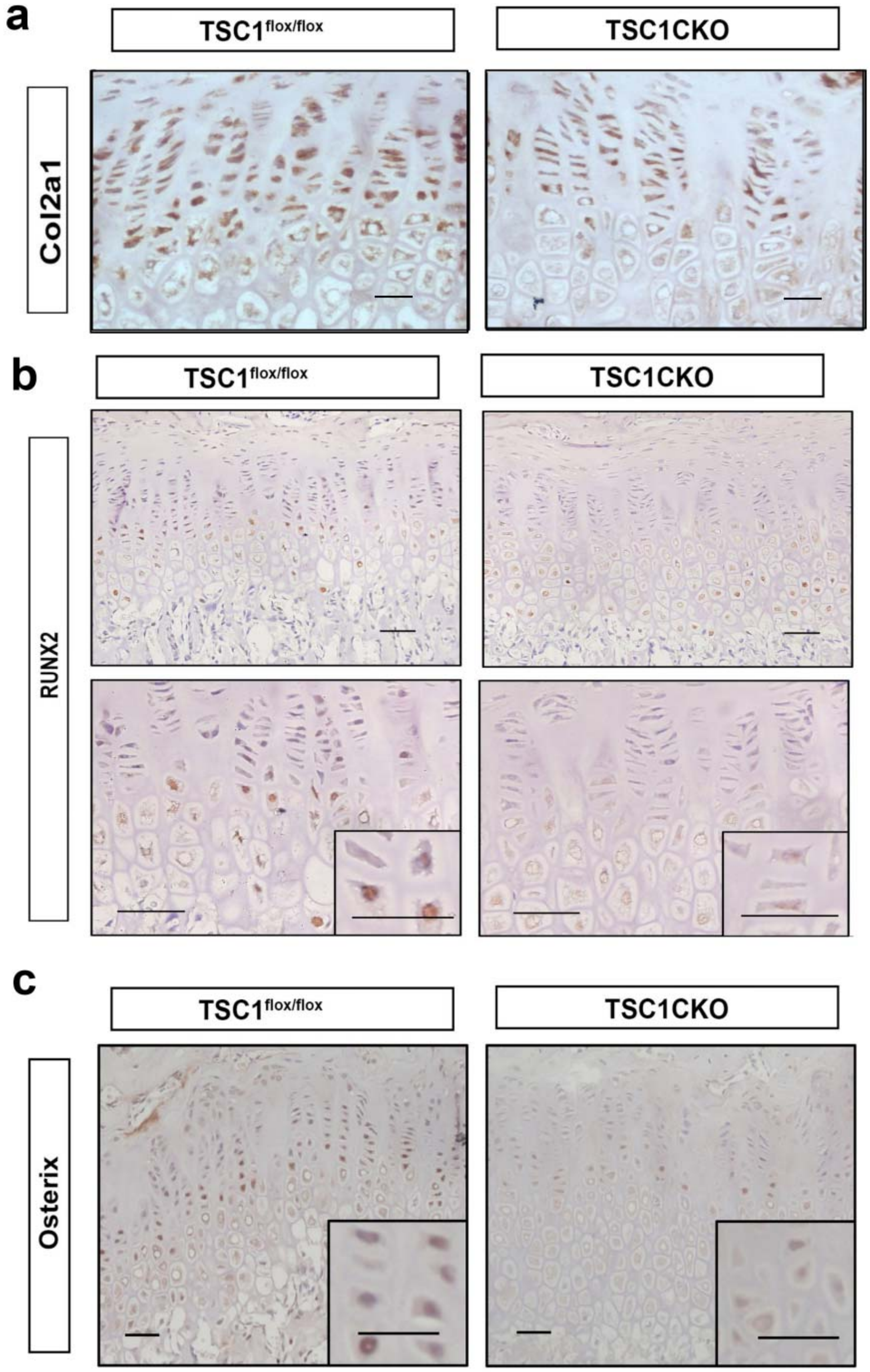

Supplementary Figure 4. Impaired chondrocyte differentiation in TSC1CKO mice. Immunohistochemical analysis of (**a**) Col2α1, (**b**) Runx2 and (**c**) Osterix expression in TSC1CKO and control mice tibia tissue at 4 weeks. Scale bar = 100 μm.

Supplementary Figure 5

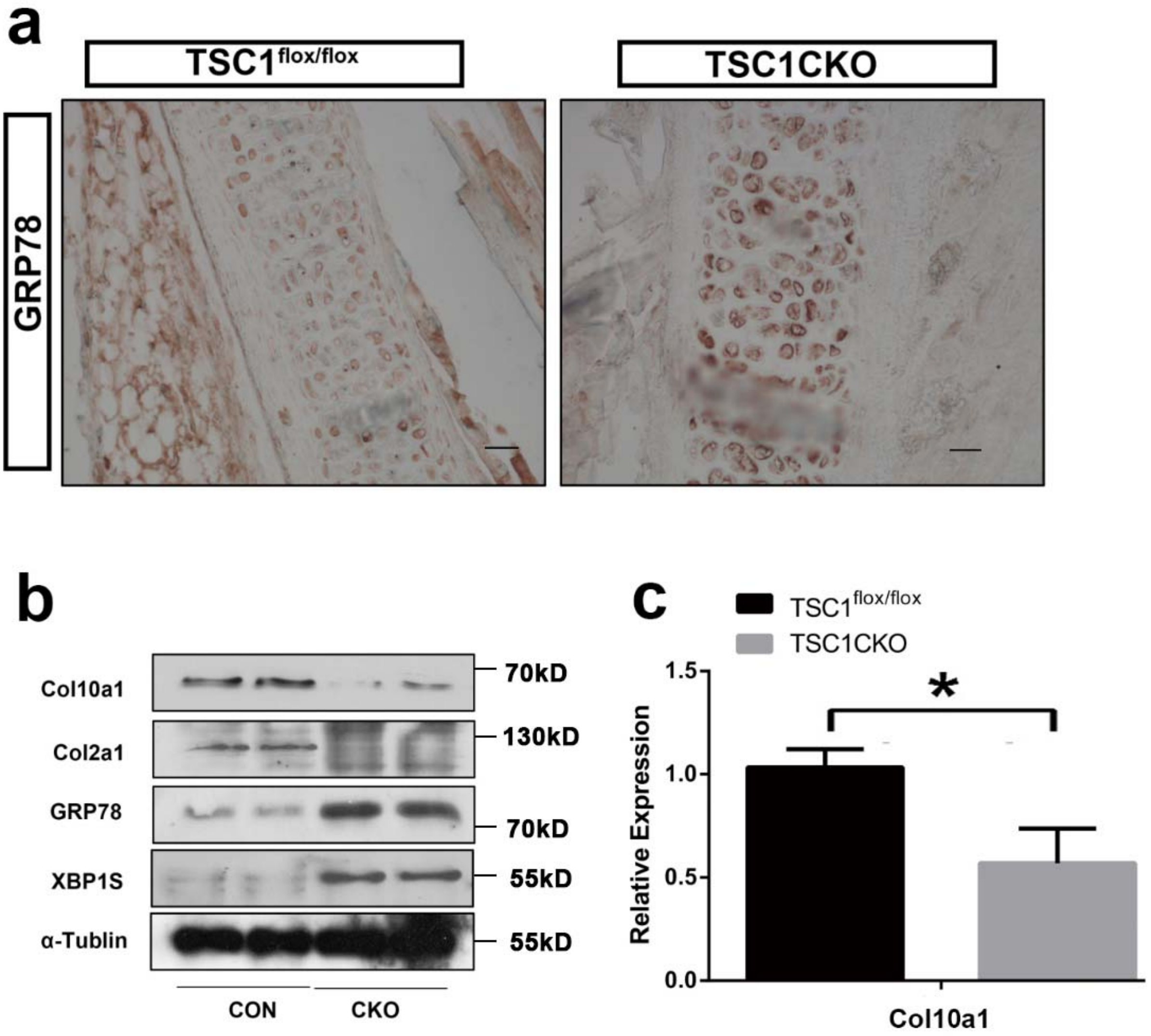

Supplementary Figure 5. Enhanced endoplasmic reticulum (ER) stress and reduced Collagen X mRNA level may contribute to the decreased Collagen X protein expression in TSC1CKO mice. (a) Immunohistochemical analysis of GRP78 in the rib cartilage of 3-week-old TSC1CKO and control mice. Scale bar = 100 μm.(b) Western blot analysis of GRP78, XBP1S, Col10a1, Col2a1 expression in cartilage of 3-week-old TSC1CKO and control mice. (c) qPCR analysis of Collagen X and Collagen II mRNA expression in growth plate of 3-week-old TSC1CKO and control mice. Student's t test, $P<0.05$, $n=4$.

Supplementary Figure 6

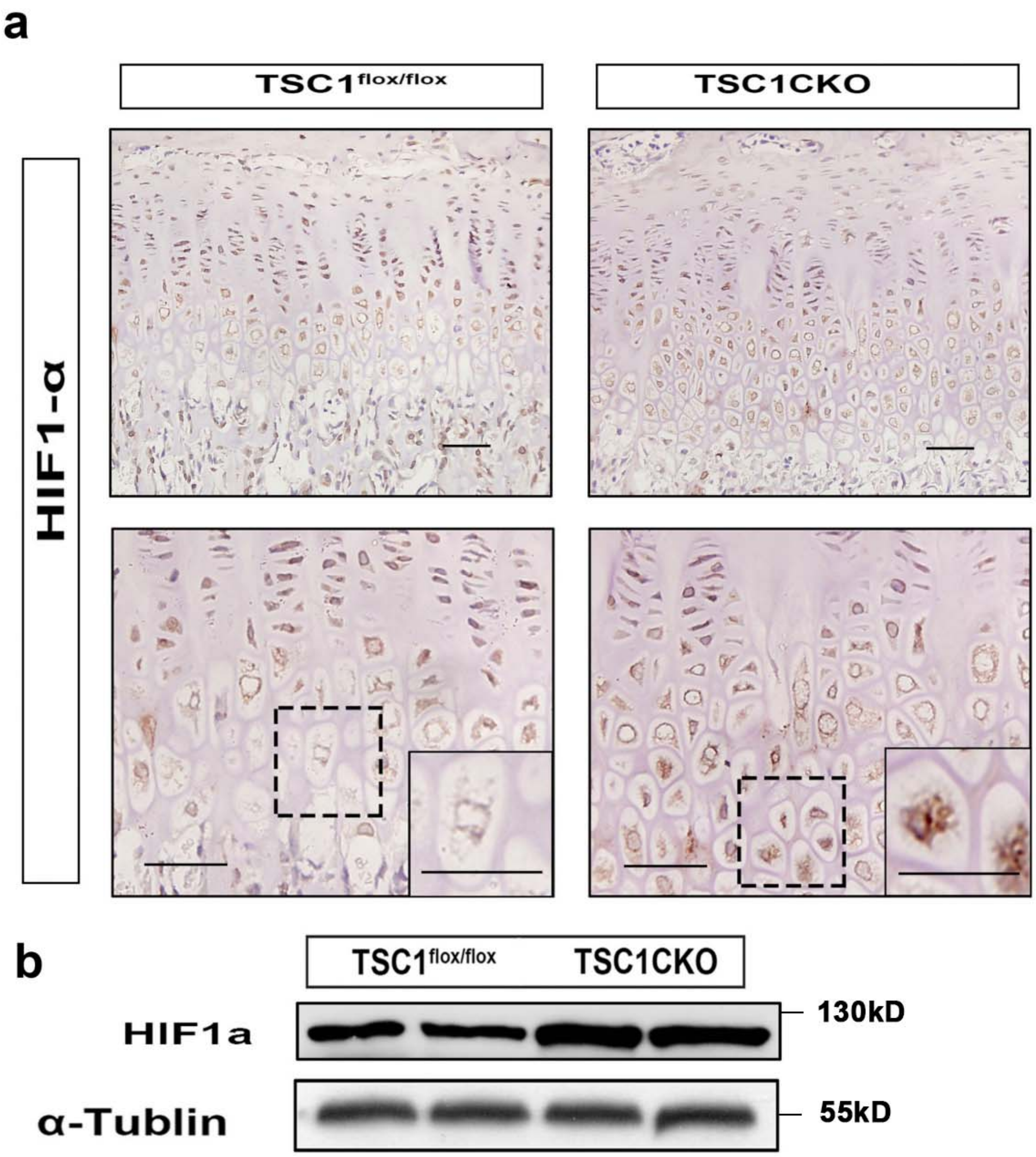

Supplementary Figure 6. Expression of HIF-1 in growth plate of TSC1CKO mice. (**a**)Immunohistochemical analysis of HIF-1α expression in TSC1CKO and control mice tibia tissue at 4 weeks. Scale bar = 100 μm. (**b**) Immunoblotting analysis of HIF-1α expression in TSC1CKO and control mice tibia tissue at 4 weeks.

Supplementary Figure 7

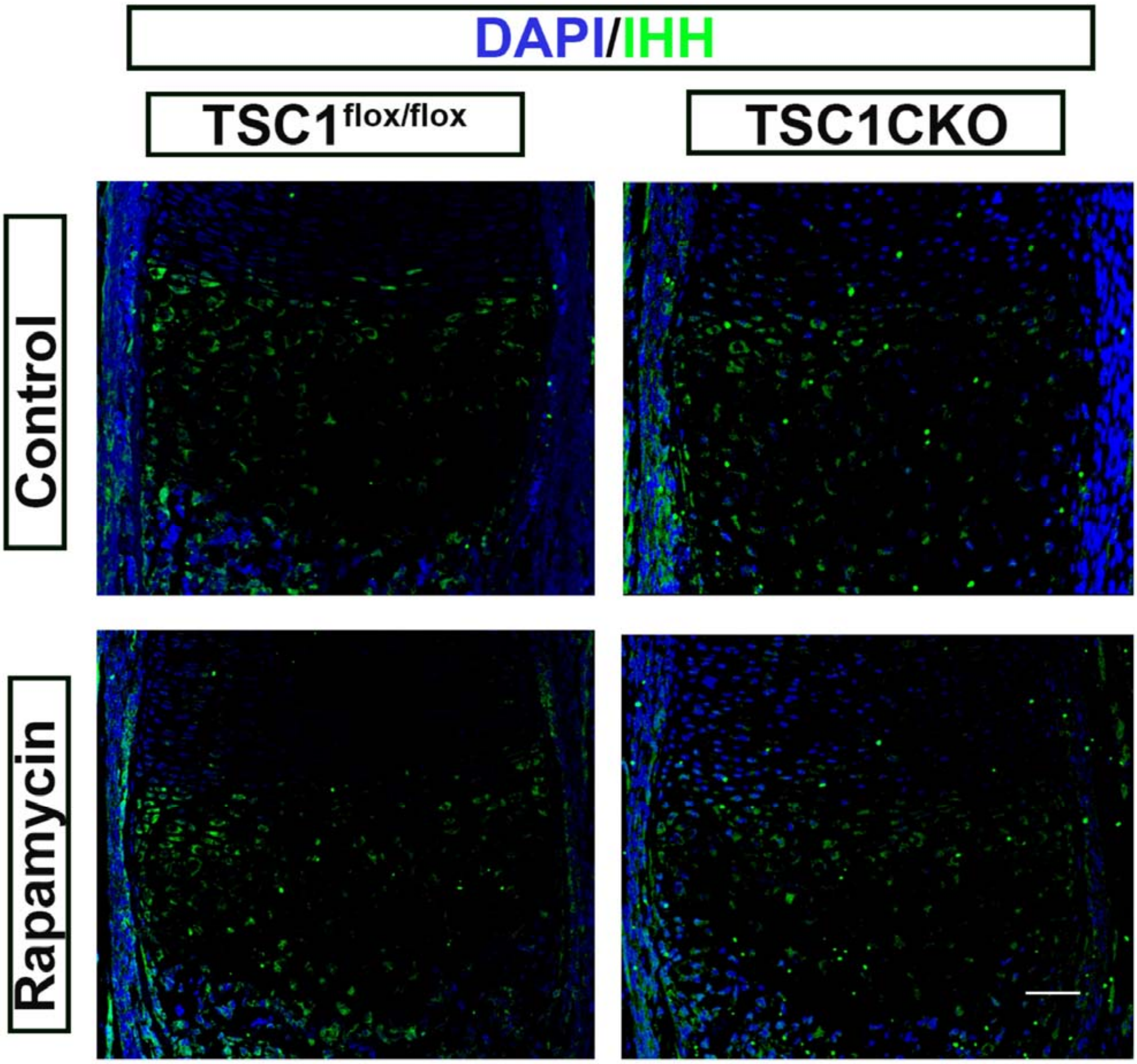

Supplementary Figure 7. Immunofluorescene of IHH expression in E16.5 mice femurs treated with rapamycin for 2 days or not. IF of IHH in E16.5 femurs showing that no change in IHH expression in $TSC1^{flox/flox}$ and TSC1CKO treated with rapamycin or not. DNA: blue, IHH: green. Scale bar = 100 μm.

Supplementary Figure 8

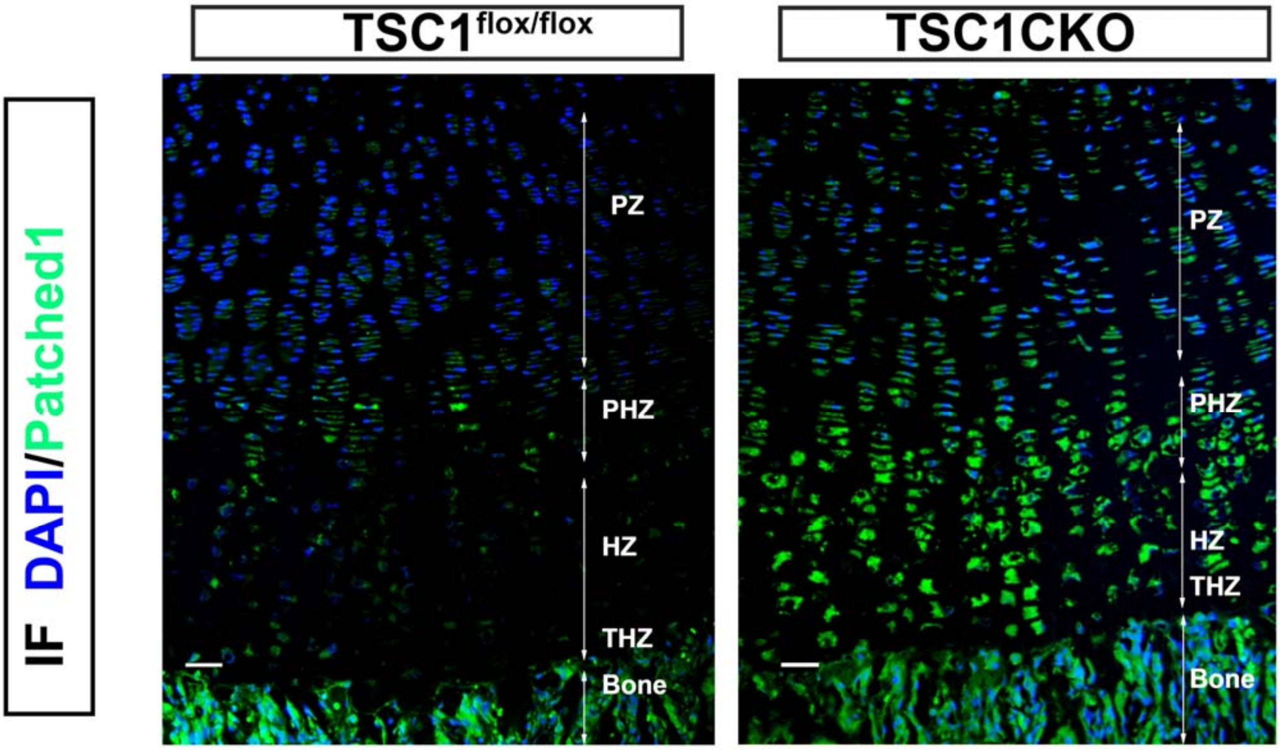

Supplementary Figure 8. Expression of Patched1 in growth plate of TSC1CKO mice. Immunofluorescence analysis of Patched1 expression in TSC1CKO and control mice tibia tissue at D10. Scale bar = 100μm.

Supplementary Figure 9

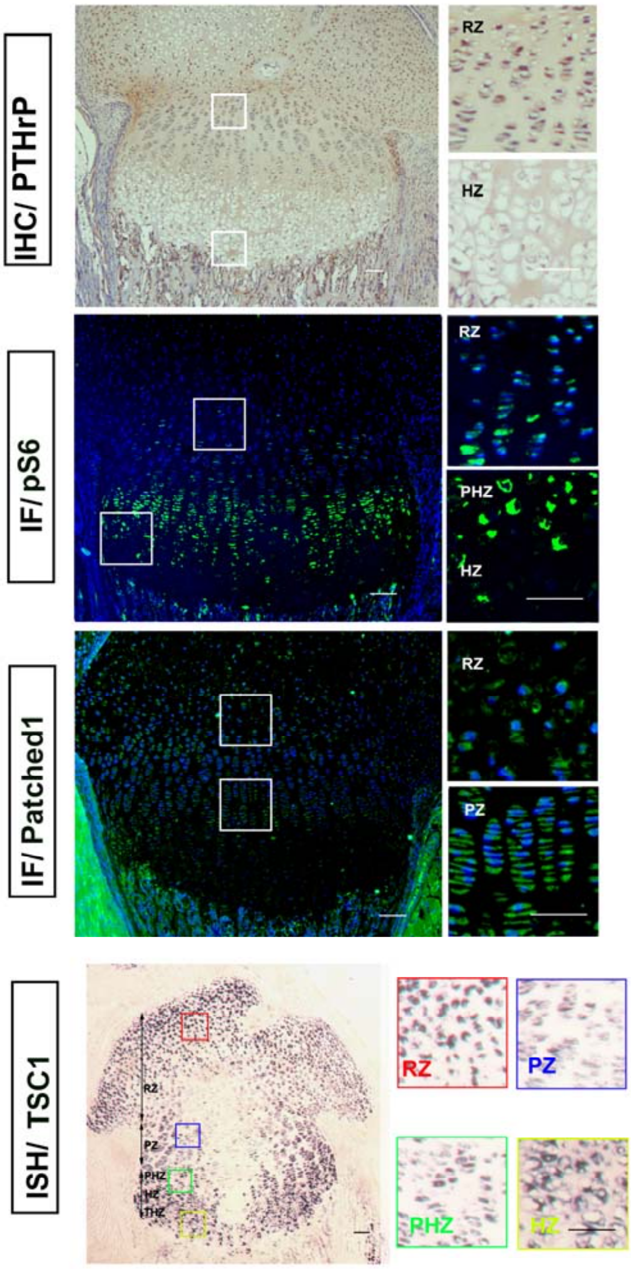

Supplementary Figure 9. Comparative expression analysis of TSC1, pS6, PTHrP and Patched1 in growth plate of 10-day- old mice. IHC analysis of PTHrP expression, IF analsysi of Patched1 and pS6 expression, and ISH analysis of TSC1 expression. Boxed areas are presented by a higher magnification on the right. Scale bar = 100 μm.

Supplementary Figure 10

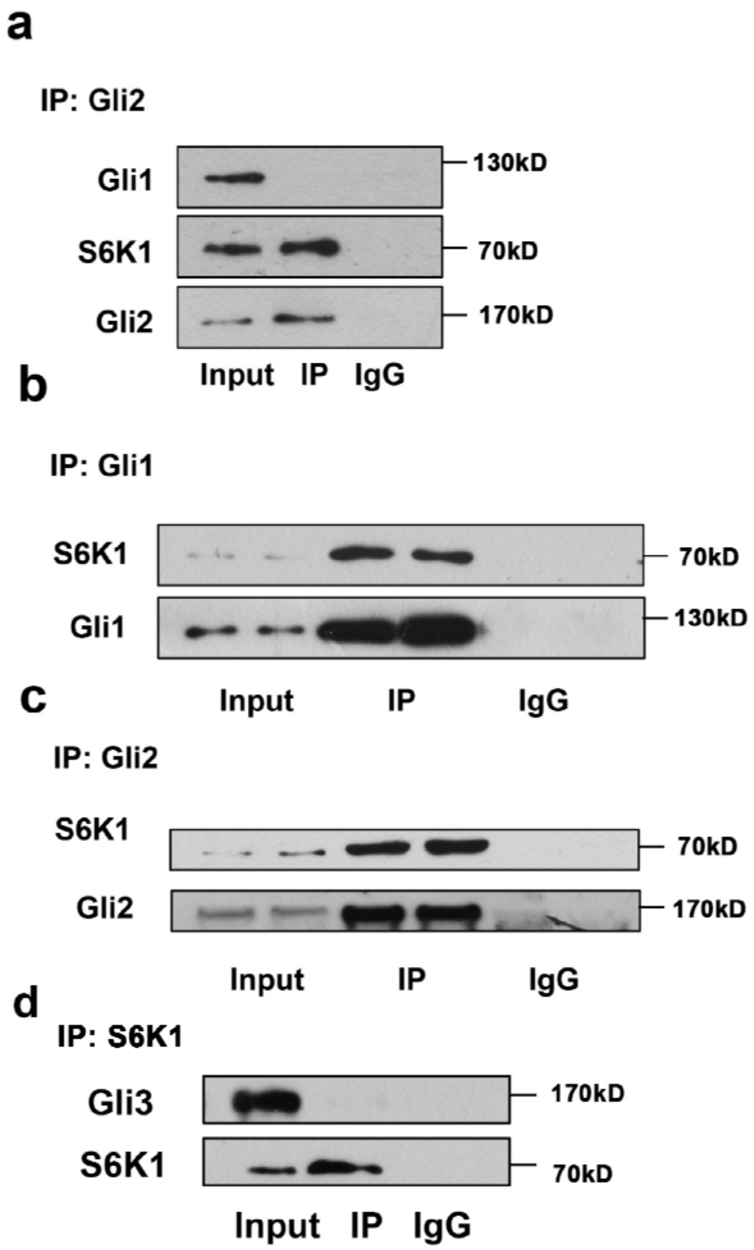

Supplementary Figure 10. Gli1 and Gli2 could not form a dimeric complex in primary cultured chondrocytes. (a) Immunoprecipitation analysis showing the interactions of Gli2 with S6K1, but not Gli1 in chondrocytes. (b) (c) Immunoprecipitation analysis showing that S6K1 could be precipitated by the antibodies against Gli1 or Gli2. (d) Immunoprecipitation analysis showing that there was no interaction between Gli3 and S6K1.

Supplementary Figure 11

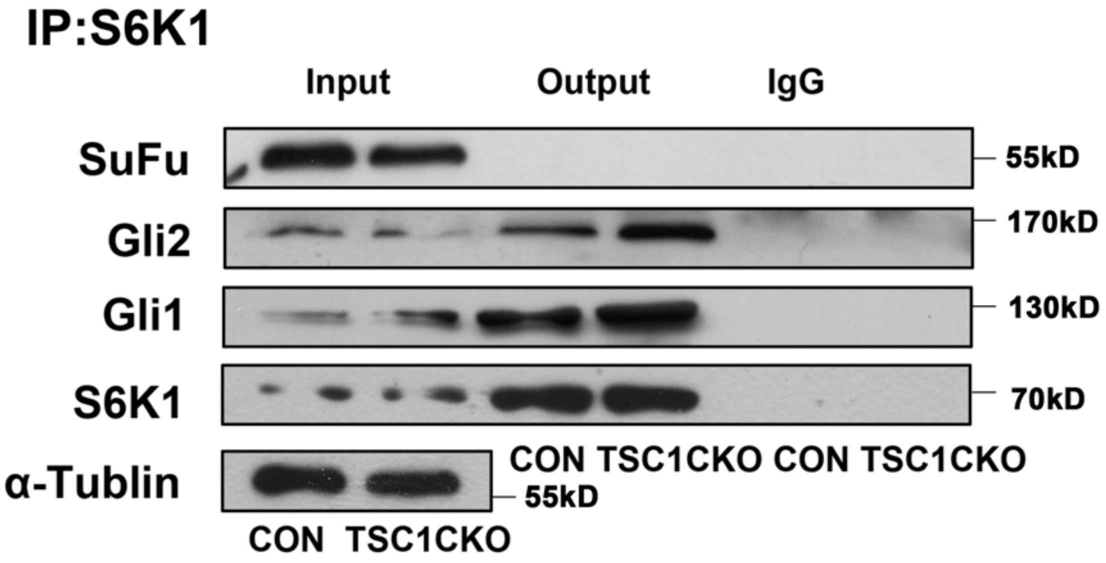

Supplementary Figure 11. p70S6K1 does not associate with SuFu in chondrocytes. Immunoprecipitation analysis showing the interactions of SuFu and Gli1/2, SuFu and S6K1 in chondrocytes from control or TSC1CKO mice.

Supplementary Figure 12a

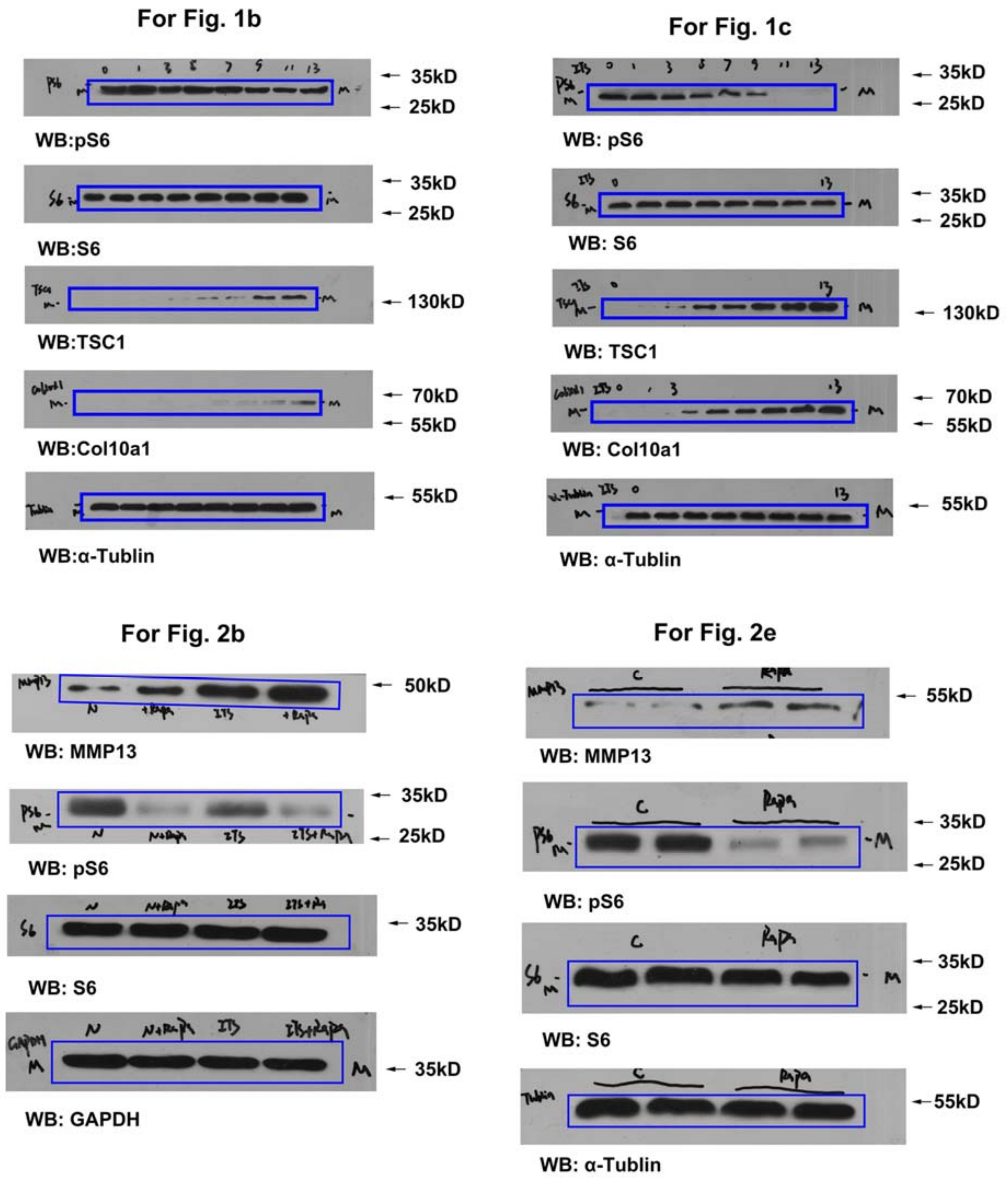

Supplementary Figure 12a. Uncropped picture of Western blots and IP results in Fig. 1b-c, Fig. 2b, 2e. Blue boxes show the cropped regions. Arrows indicate the size marker.

Supplementary Figure 12b

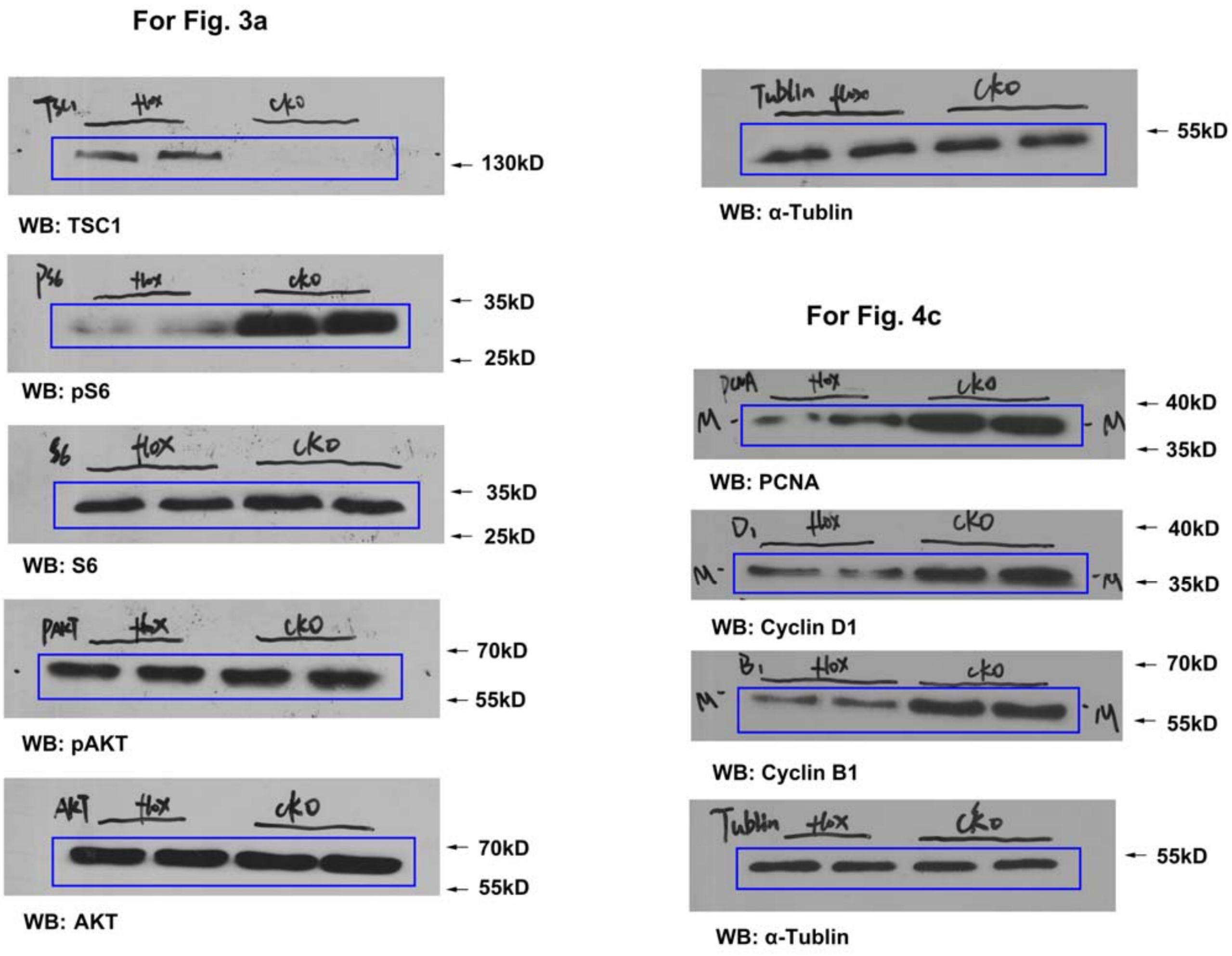

Supplementary Figure 12b. Uncropped picture of Western blots and IP results in Fig. 3a, 4c.

Blue boxes show the cropped regions. Arrows indicate the size marker.

Supplementary Figure 12c

For Fig. 5b

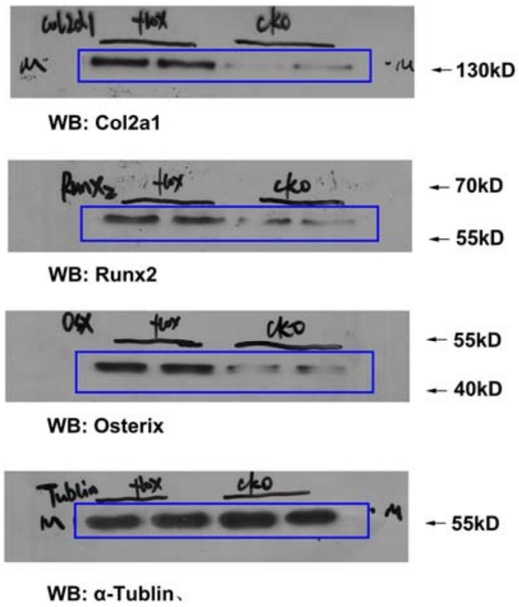

For Fig. 5d

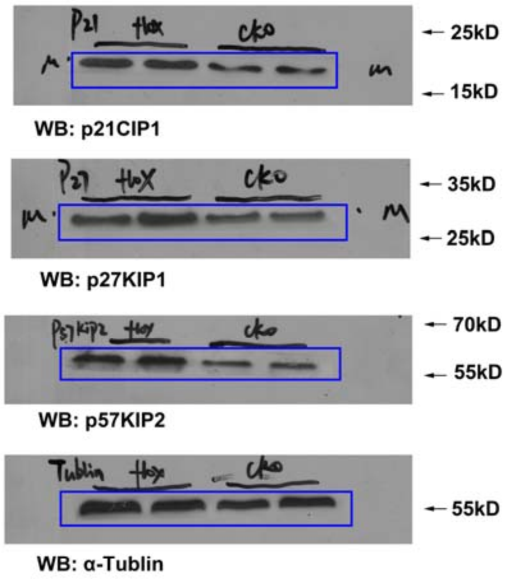

For Fig. 5f

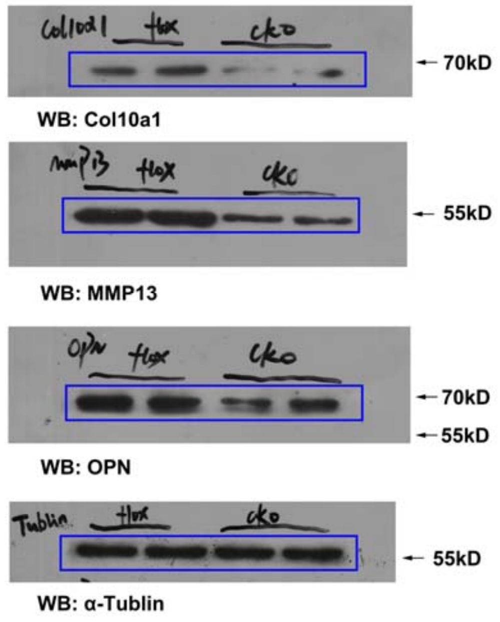

For Fig. 6f

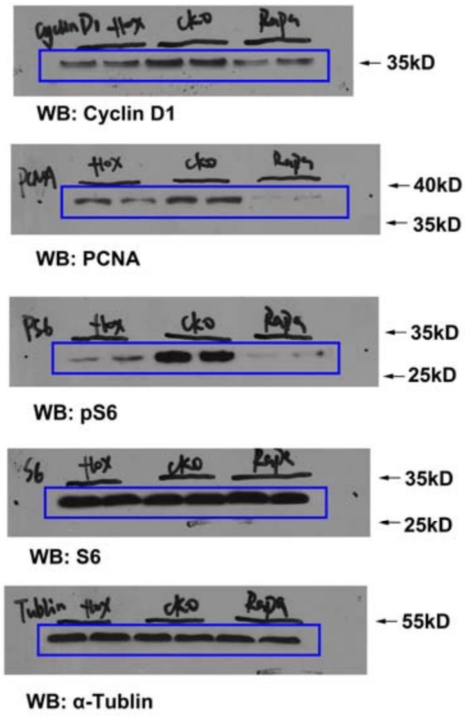

Supplementary Figure 12c. Uncropped picture of Western blots and IP results in Fig. 5b, 5d, 5f, 6f. Blue boxes show the cropped regions. Arrows indicate the size marker.

Supplementary Figure 12d

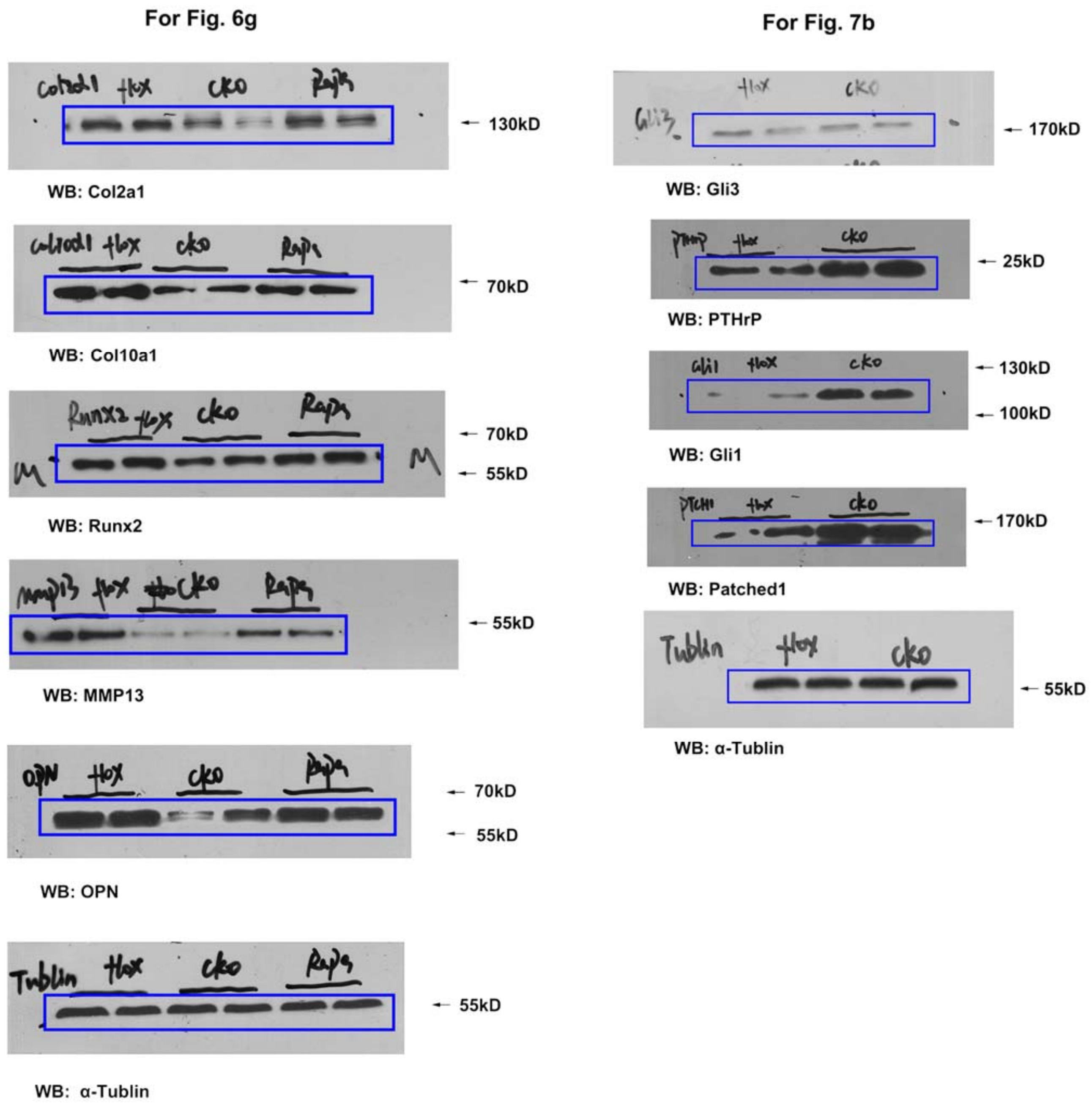

Supplementary Figure 12d. Uncropped picture of Western blots and IP results in Fig. 6g, 7b. Blue boxes show the cropped regions. Arrows indicate the size marker.

Supplementary Figure 12e

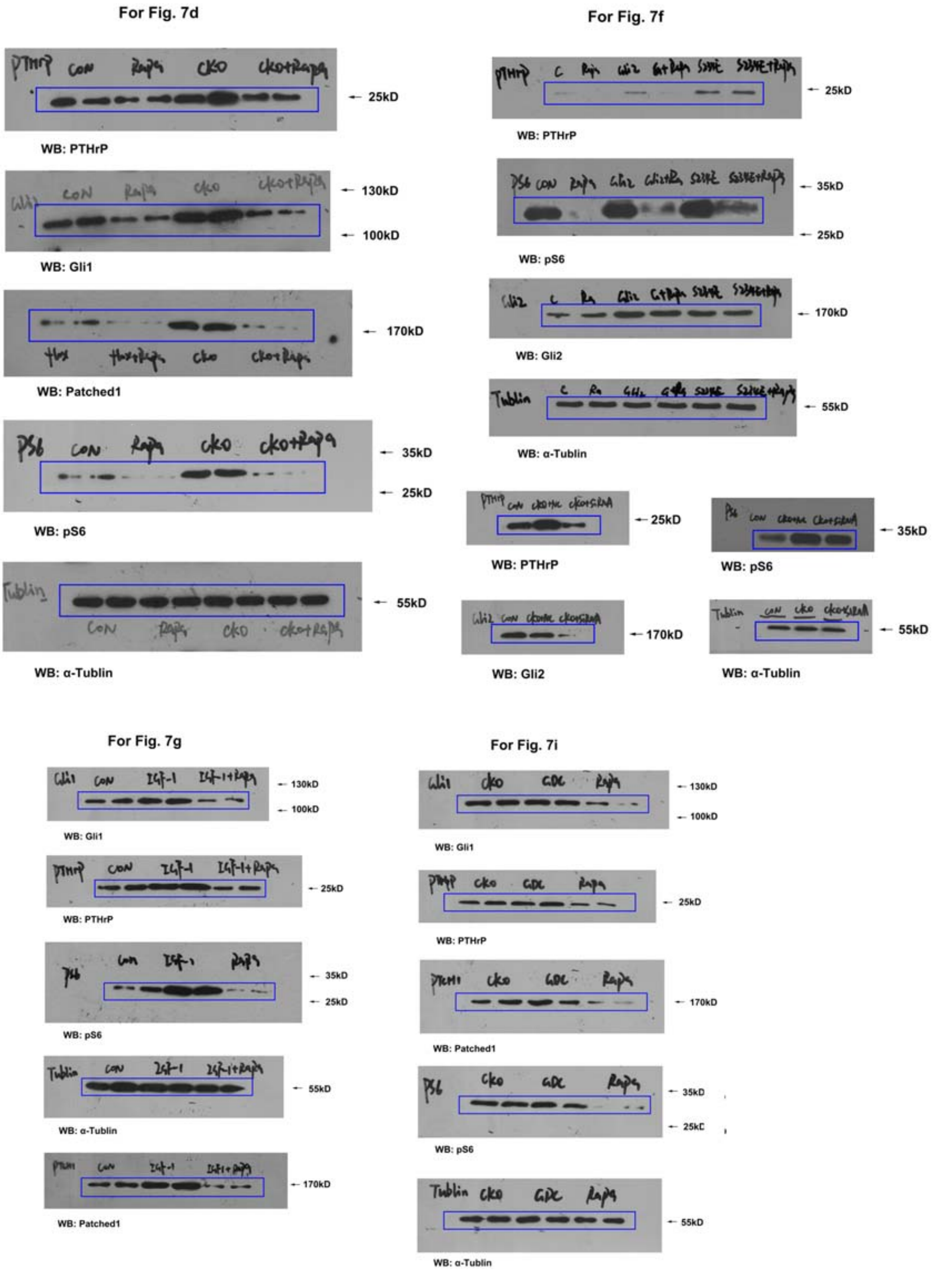

Supplementary Figure 12e. Uncropped picture of Western blots and IP results in Fig. 7d, 7f, 7g, 7i. Blue boxes show the cropped regions. Arrows indicate the size marker.

Supplementary Figure 12f

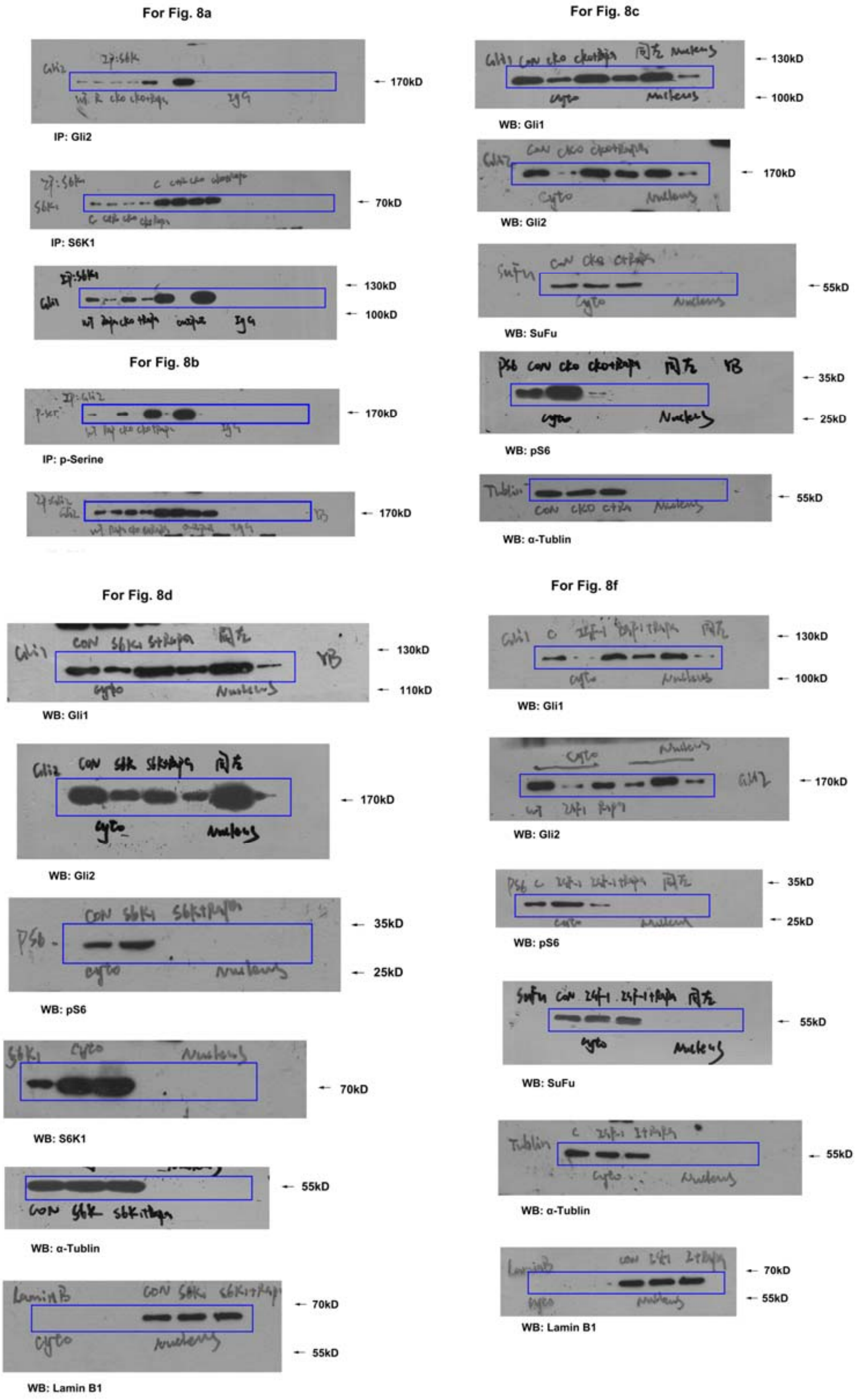

Supplementary Figure 12f. Uncropped picture of Western blots and IP results in Fig. 8a, 8c, 8d, 8f. Blue boxes show the cropped regions. Arrows indicate the size marker.

Supplementary Figure 12g

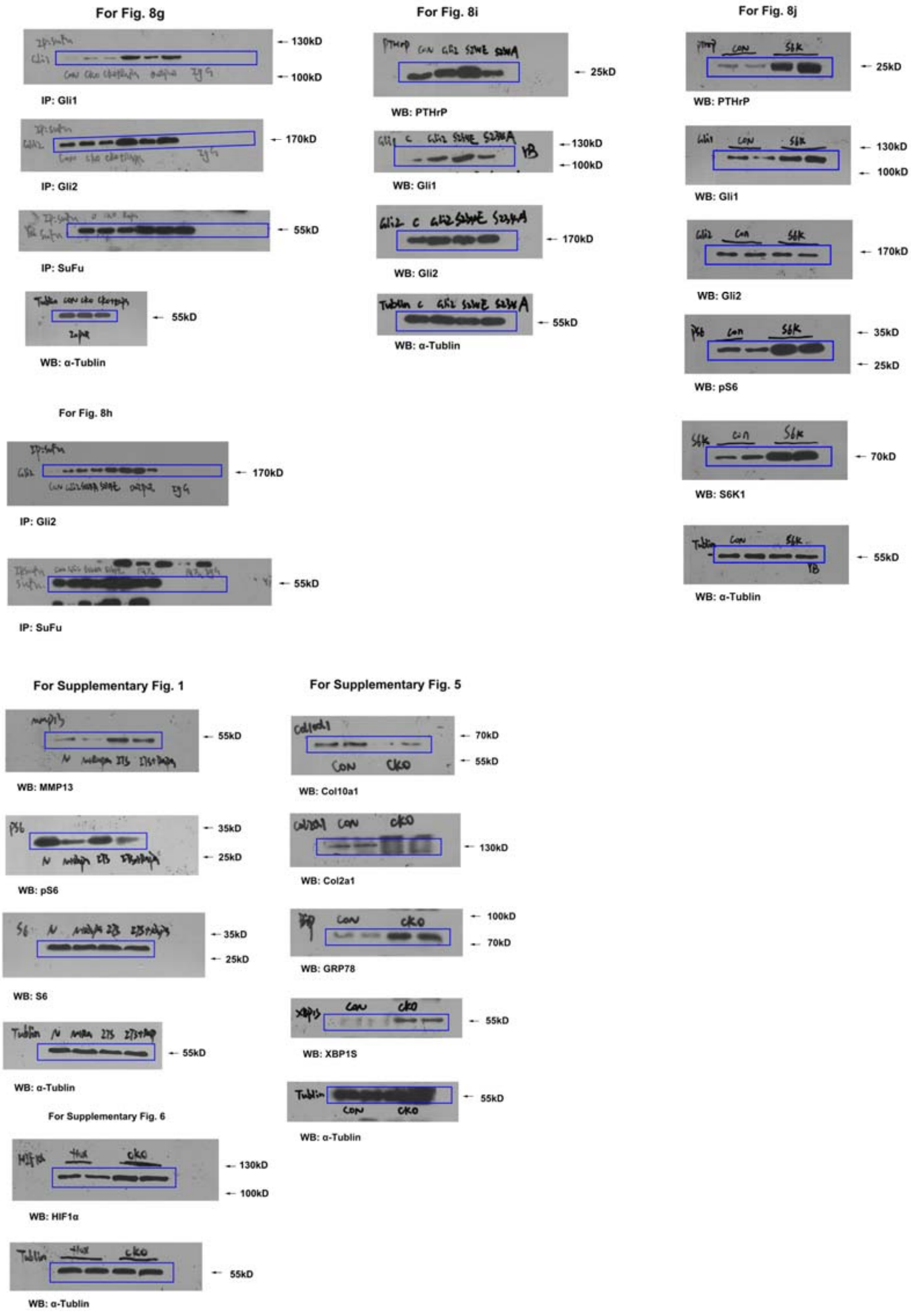

Supplementary Figure 12g. Uncropped picture of Western blots and IP results in Fig. 8g, 8i, 8j, Supplementary Figure 1, 5, 6. Blue boxes show the cropped regions. Arrows indicate the size marker.

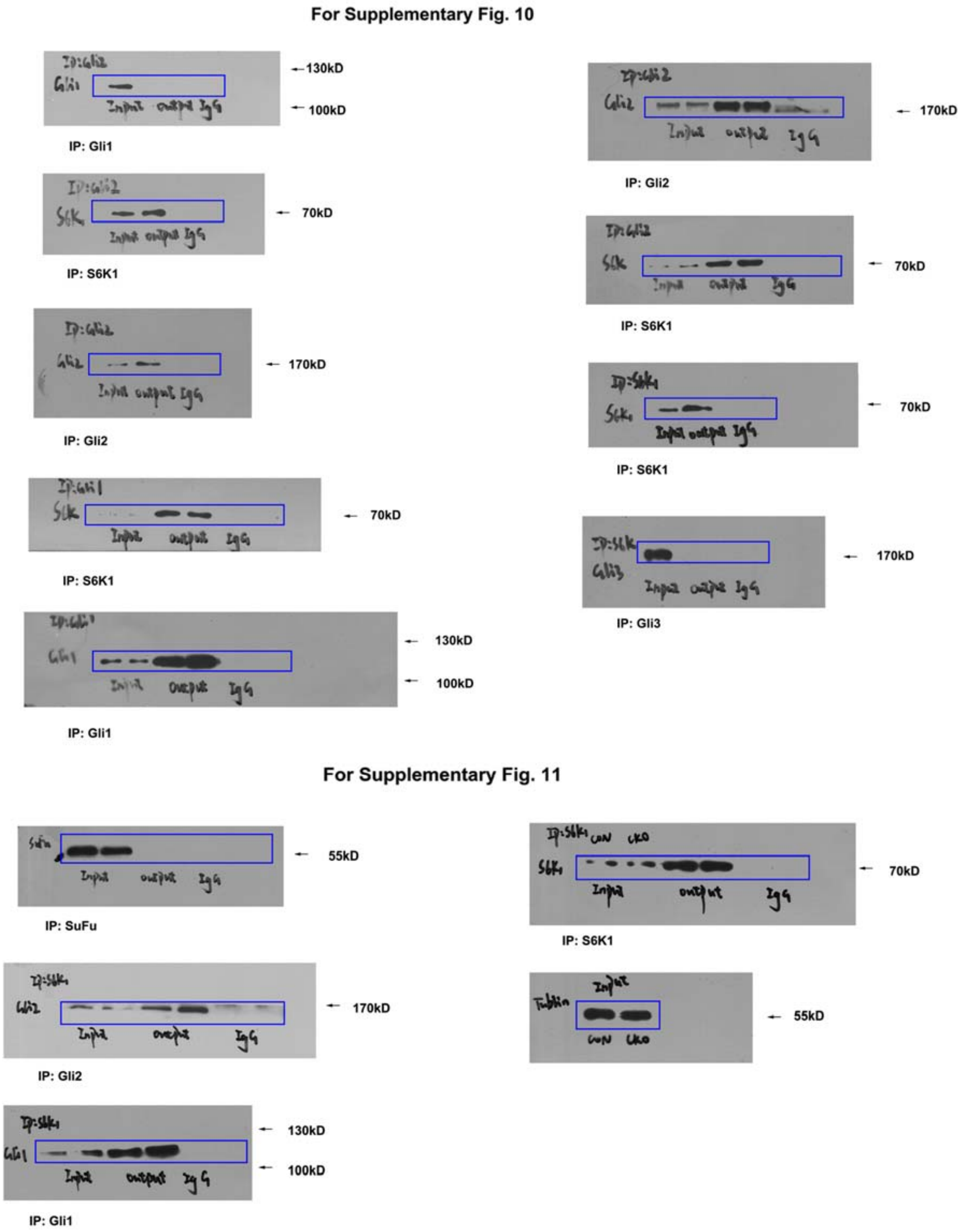

Supplementary Figure 12h. Uncropped picture of Western blots and IP results in Supplementary Figure 10, 11. Blue boxes show the cropped regions. Arrows indicate the size marker.

Supplementary Figure 12i

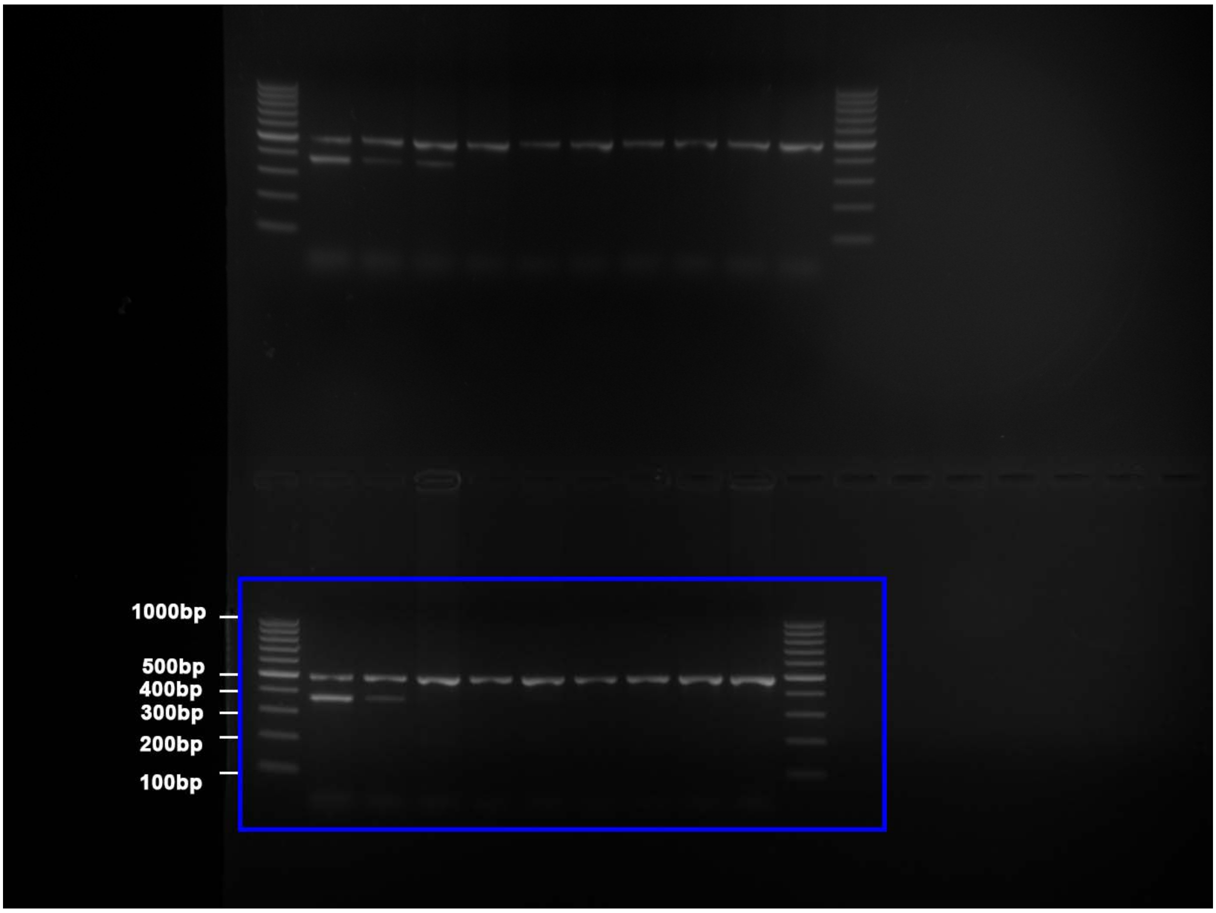

Supplementary Figure 12i. Uncropped picture of agarose gel electrophoresis in Supplementary Figure 2.

Supplementary Table

Supplementary Table 1 Primers used in this study

Sequence Name	Sequence (5' to 3')
**Genotyping primers**	
TSC1 loxp (forward)	GTCACGACCGTAGGAGAAGC
TSC1 loxp (reverse)	GAATCA ACCCCACAGAGCAT
Col2a1 (forward)	GCCTGCATTACCGGTCGATGC
Col2a1 (reverse)	CAGGGTGTTATAAGCAATCCC
**q-PCR primers**	
Col2a1 (forward)	CTGGTGGAGCAGCAAGAGCAA
Col2a1 (reverse)	CAGTGGACAGTAGACGGAGGAAAG
Col10a1 (forward)	GCAGCATTACGACCCAAG AT
Col10a1 (reverse)	CAT GAT TGCACTCCCTGAAG
MMP13 (forward)	CAGTTGACAGGCTCCGAGAA
MMP13 (reverse)	CGTGTGCCAGAAGACCAGAA
IHH (forward)	CTCTTGCCTACAAGCAGTTCA
IHH (reverse)	CCGTGTTCTCCTCGTCCTT
PTHrp (forward)	CAGTGGAGTGTCCTGGTATT
PTHrp (reverse)	GATCTCCGCGATCAGATGGT
Gli1(forward)	CCAAGCCAACTTTATGTCAGGG
Gli1(reverse)	AGCCCGCTTCTTTGTTAATTTGA
Patched1(forward)	AAAGAACTGCGGCAAGTTTTTG
Patched1(reverse)	CTTCTCCTATCTTCTGACGGGT
GAPDH (forward)	TGGCCTTCCGTGTTCCTAC
GAPDH (reverse)	GAGTTGCTGTTGAAGTCGCA
**cDNA probe cloning Primer**	
Col10a1 (forward)	CCGCTCGAGTGATGGACCTGCTGGCTCT
Col10a1 (reverse)	TTCTCCTGGGATACCTGGCTGA
Tsc1(forword)	CAAGCTTGCGGCTCTGGAGGAACACAATG

Tsc1(reverse)	CGGATCCTCACTGCTGCTGCTGCTGCT
MMP13(forward)	CCGCTCGAGCCACAGTTGACAGGCTCCGAGA
MMP13(reverse)	CCGCTCGAGGGGATAGGGCTGGGTCACACTT
**Promoters cloning Primers**	
Patched1 (forward)	GGCTAGCATCACGAGCAGGCCGAAGACA
Patched1 (reverse)	AATTGATATCCGCCTTCCATTGCCACATTGC
PTHrp (forward)	GGGGTACCGCAAAGAATCGGAGGCTGACACT
PTHrp (reverse)	CCCAAGCTTCTCTCTCAAACCGCACAGGCAAA
**Mutation primers**	
S234A-F	CAAGCGGGCGCTGGCCATCTCCCCACTCTC
S234A-R	GAGAGTGGGGAGATGGCCAGCGCCCGCTTG
S234E-F	CAAGCGGGCGCTGGAAATCTCCCCACTCTC
S234E-R	GAGAGTGGGGAGATTTCCAGCGCCCGCTTG
**CHIP qPCR primers**	
PTHrp promoter-F	CCAACCCTTCCCAAACAGTAAG
PTHrp promoter-R	GGACTAAACCTAGCACCCAGAAGA
